# Characterization of HIV-induced remodeling reveals differences in infection susceptibility of memory CD4^+^ T cell subsets *in vivo*

**DOI:** 10.1016/j.celrep.2021.109038

**Published:** 2021-04-27

**Authors:** Guorui Xie, Xiaoyu Luo, Tongcui Ma, Julie Frouard, Jason Neidleman, Rebecca Hoh, Steven G. Deeks, Warner C. Greene, Nadia R. Roan

**Affiliations:** 1 Gladstone Institute of Virology, San Francisco, CA 94158, USA; 2 Department of Urology, University of California, San Francisco, San Francisco, CA 94158, USA; 3 Division of HIV, Infectious Diseases and Global Medicine, University of California San Francisco, San Francisco, CA 94110, USA; 4 Departments of Medicine and Microbiology and Immunology, University of California, San Francisco, San Francisco, CA 94158, USA; 5 Lead contact

## Abstract

Relatively little is known about features of T cells targeted by HIV *in vivo*. By applying bioinformatics analysis to mass cytometry (CyTOF)-phenotyped specimens from individuals with viremia and *in-vitro*-infected cells from uninfected donors, we provide an atlas of the phenotypes of *in vivo* and *in vitro* HIV-susceptible cells. T helper 17 (Th17) and α4β1^+^ cells are preferentially targeted *in vivo*, whereas T effector memory (Tem), T transitional memory (Ttm), Th1, and Th1/Th17 subsets are targeted *in vitro*. Multiple proteins—including chemokine and cytokine receptors—are remodeled by HIV *in vivo*, and these changes are mostly recapitulated *in vitro*. HIV remodels cells to a T follicular helper (Tfh) phenotype. Using clustering, we uncover a subset of CD29-expressing, Tem-like cells that are highly susceptible to infection *in vivo* and *in vitro* and experimentally confirm that susceptibility. These studies provide an in-depth look at features of HIV-susceptible cells in individuals with viremia and demonstrate that some—but not all—HIV-susceptible cells identified *in vitro* effectively model *in vivo* susceptibility.

## In brief

Xie et al. provide an in-depth view of the features of uninfected, HIV-infected, and bystander cells from individuals with viremia as compared with *in vitro* HIV infection. By applying bioinformatics approaches on CyTOF datasets, they describe the features of HIV-susceptible cells and identify surface receptors remodeled during *in vivo* infection.

## Graphical abstract

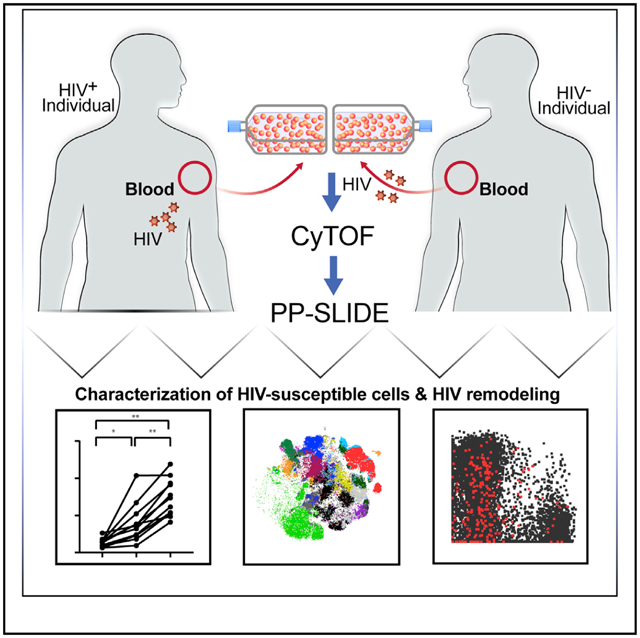

## INTRODUCTION

Multiparameter analysis of single cells using a growing array of “-omics” technological and analytical approaches has over the past 10 years revolutionized immunology research by providing an unprecedented high definition of individual cells. One such technology has been mass cytometry (CyTOF), which uses metal-labeled antibodies to simultaneously quantitate the levels of ~40 different surface and intracellular proteins on individual cells. Although not as unbiased or high-parametric as single-cell RNA sequencing (scRNA-seq), CyTOF allows for characterization of cells at the protein level, which better reflects cellular function. Furthermore, its relatively high-throughput capability enables characterization of rare populations of cells without the need for extensive pre-purification. In addition to providing a high-resolution “picture” of immune cells, the high-dimensional nature of CyTOF datasets has enabled implementation of various pseudotime analytical approaches that place individual cells along developmental trajectories (Bendall et al., 2014).

The ability of pseudotime approaches to trace cells in a perturbed system back to cells in a pre-perturbation state is particularly advantageous for the study of infection by HIV and other viruses that extensively remodel the host cells they infect (Cavrois et al., 2017; Ma et al., 2020; Sen et al., 2014). Remodeling of CD4^+^ T cells by HIV includes downregulation of cell-surface CD4 and CD28 and upregulation of select homing receptors upon infection (Garcia and Miller, 1991; Ma et al., 2020; Swigut et al., 2001). HIV-induced remodeling makes it challenging to determine whether differential features of infected cells result from preferential infection of cells harboring those features, HIV-induced changes via remodeling, or a combination of both processes. To address that issue, we previously developed a method, called predicted precursor, as determined by single-cell linkage for distance estimation (PP-SLIDE), which adapted a prior method quantitating viral-induced remodeling (Sen et al., 2014) to predict the original features of HIV-susceptible cells before HIV-induced remodeling (Cavrois et al., 2017). PP-SLIDE takes advantage of the fact that, despite HIV-induced remodeling, enough of the original (pre-infection) phenotypic features of the infected cell are retained in a manner that can be captured by high-dimensional analyses of CyTOF datasets. *In vitro* infection experiments implementing PP-SLIDE entailed mock treating or infecting a diverse population of primary cells with HIV and then phenotyping the cells by CyTOF. By matching every HIV-infected remodeled cell to the corresponding “atlas” of uninfected (UI) cells from the mock-treated sample, the likely original phenotypes of cells targeted for HIV infection were identified. This approach was previously implemented on tonsillar and endometrial CD4^+^ T cells infected *in vitro* with a CCR5-tropic reporter HIV and was validated through a variety of sorting experiments and functional assays (Cavrois et al., 2017; Hsiao et al., 2020; Ma et al., 2020; Neidleman et al., 2020).

However, few studies have analyzed the phenotypes of cells infected by HIV *in vivo*. One reason is the technical challenge of unambiguously identifying those cells in specimens from individuals with viremia. Because these cells tend to be rare, relative to the infected cells resulting from *in vitro* infection, their identification using the common fluorescence-activated cell sorting (FACS) approach of staining for intracellular Gag is ineffective because of a low signal-to-noise ratio. This problem was overcome recently by staining *in-vivo*-infected cells simultaneously for HIV RNA and Gag expression (Baxter et al., 2016) or using two different anti-Gag antibodies (Pardons et al., 2019) during FACS analysis. Such dual-staining approaches overcame the signal-to-noise issue and were used to characterize, by FACS, the phenotypes of infected cells from patients with viremia, as well the phenotypes of reactivated cells after *ex vivo* stimulation of cells from virally suppressed patients (Baxter et al., 2016; Pardons et al., 2019). However, these studies did not address the problem of post-infection cell remodeling.

Here, we characterize, by CyTOF, the phenotypes of *in vivo* HIV-infected cells by dually labeling peripheral blood mononu-clear cells (PBMCs) from viremic HIV-infected individuals with two metal-conjugated anti-Gag antibodies and implementing PP-SLIDE on the CyTOF datasets to trace the remodeled, HIV-infected cells to their predicted pre-infection phenotypes. This was accomplished by analyzing paired longitudinal specimens of infected individuals when they were viremic, versus when they were virally suppressed on antiretroviral therapy (ART). PP-SLIDE enabled us to assess not only what cells were preferentially targeted for HIV infection *in vivo* but also to what extent various proteins were remodeled during *in vivo* infection. Finally, by conducting a parallel set of studies, in which PP-SLIDE analysis was implemented on *in-vitro*-infected PBMCs, we were able to compare and contrast the features of HIV-susceptible cells *in vitro* versus *in vivo* and to dissect which remodeling features that occur *in vitro* also manifest *in vivo*.

## RESULTS

### Comparison of *in vitro* and *in vivo* UI, HIV-infected, and bystander T cells

**Figure 1.**
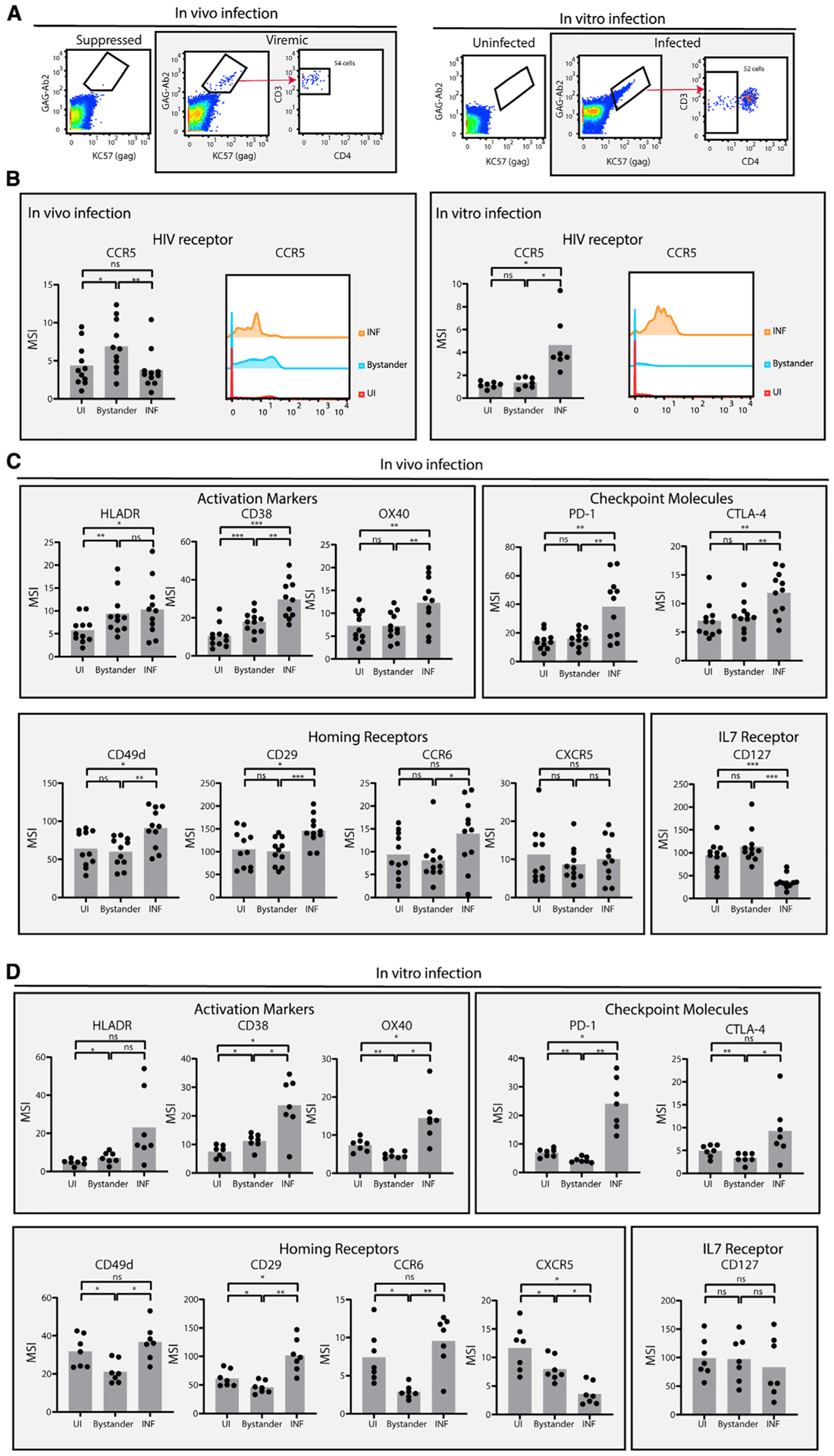
Characterization of *in vivo* and *in vitro* uninfected, bystander, and HIV-infected cells (A) HIV-infected T cells were defined as CD3^+^CD8^−^ cells that bound both sets of anti-Gag antibodies (KC57 and Gag-Ab2, a mix of the Gag antibody clones indicated in the STAR Methods) and that had downregulated cell-surface CD4. (Left) CD3^+^CD8^−^ cells from the same individual (donor 2043) at a virally suppressed time point versus a viremic time point, showing CD4 downregulation on infected cells from the viremic time point. (Right) CD3^+^CD8^−^ cells from mock-treated (“Uninfected” [UI]) versus HIV-exposed (“Infected”) PBMC cultures, showing gating on CD4-downregulated cells among the Gag-expressing cells. The remaining *in vivo* and *in vitro* specimens are presented in Figure S1. (B) CCR5 is highly expressed on *in vitro*, but not *in vivo*, HIV-infected cells. Shown are the mean signal intensity (MSI) levels of CCR5 among UI, bystander (Gag^−^ cells from viremic specimens or *in-vitro*-infected cultures), and infected (INF) CD3^+^CD8^−^ cells, displayed as bar graphs showing the individual specimens or as a histogram showing cells combined from all the specimens. (C and D) Levels of activation markers (HLADR, CD38, and OX40), checkpoint molecules (PD-1, CTLA-4), homing receptors (CD49d, CD29, CCR6, and CXCR5) and the alpha chain of the IL-7 receptor (CD127) were compared among UI, bystander, and INF cells from the *in vivo* (C) or *in vitro* (D) specimens. The remaining antigens not shown here are presented in Figure S3. *p < 0.05, **p < 0.01, ***p < 0.001 as determined by a Student’s paired t test and adjusted for multiple testing using the Benjamini-Hochberg for false-discovery rate (FDR); ns, not significant. See also Figures S1–S3.

A CyTOF panel was designed to phenotype HIV-infected CD4^+^ T cells (STAR Methods). This panel included two different sets of anti-Gag antibodies labeled with different metal isotopes to enable detection of the rare *in-vivo*-infected cells. PBMCs from 11 HIV-infected participants were used in this study. For each participant, we obtained two specimens: one from a time point during which the participant was viremic and off ART, and the other, when the participant was virally suppressed on ART (STAR Methods). Cells from the virally suppressed time point were used as a source of patient-matched cells lacking productively infected cells because pre-infection specimens from the patients were not available and are, in general, difficult to procure. Cryopreserved cells from these specimens were revived, labeled with the CyTOF phenotyping panel, run on a mass cytometer, and analyzed for the presence of infected cells. Although the frequencies of infected (Gag^+^) cells were negligible in the suppressed time points, they were readily apparent in the viremic samples (Figures 1A and S1). The infected cells expressed high levels of CD3, but low levels of CD4, consistent with downregulation of cell-surface CD4 by HIV in the infected cells.

For comparison, we also infected PBMCs from seven HIV-seronegative donors with a CCR5-tropic HIV expressing the transmitted/founder (T/F) Env109FPB4 (Cavrois et al., 2017; Ma et al., 2020). To better match the *in vivo* specimens, the PBMCs were not stimulated with a mitogen before infection because stimulation markedly alters the phenotypes of the cells, making direct comparison to the *in vivo* samples challenging. Instead, cells were exposed to concentrated viral stocks to achieve infection rates that were sufficient for deep phenotypic analysis. Analysis of the *in vitro* specimens revealed a clear population of infected cells that were absent in the UI control (Figures 1A and S1). Only a subset of those cells had downregulated CD4. Interestingly, relative to their CD4^high^ counterparts, the *in vitro* HIV-infected cells that had downregulated CD4 were enriched among T effector memory (Tem) cells and under-represented among T central memory (Tcm) cells, and differentially expressed multiple markers within our CyTOF panel (Figure S2). To match as closely as possible the *in-vitro*- and *in-vivo*-infected cells, for all subsequent analyses, we restricted our analyses of infected cells to those with low levels of CD4, which are, likely, cells at a later stage in the HIV infection cycle.

We first conducted a systematic comparison of UI, infected, and bystander cells. All populations were pre-gated on CD3^+^CD8^−^ cells to include infected cells that had downregulated cell-surface CD4. *In-vitro*-UI cells were defined as cells in the UI culture, whereas *in-vivo*-UI cells were defined as cells in the specimen from the suppressed time point. *In vitro* bystander cells were cells in the infected culture that were not Gag^+^, whereas *in vivo* bystander cells were cells in the viremic specimen that were not Gag^+^. *In vitro*, mean expression levels of the main HIV co-receptor CCR5 were higher in infected, than UI, cells, consistent with selection of high-CCR5-expressing cells by the T/F virus. Interestingly, however, CCR5 levels were equivalent between the UI and infected cells in the *in vivo* specimens (Figure 1B). *In vivo* bystander cells, interestingly, expressed the highest levels of CCR5, whereas *in vitro* bystander cells expressed low levels of the co-receptor.

Expression levels of activation markers, checkpoint molecules, and homing receptors were also compared within the *in vivo* and *in vitro* cell populations (Figures 1C and 1D). In both sets of specimens, the activation markers human leukocyte antigen-DR iso-type (HLA-DR), CD38, and O×40 and the checkpoint/activation antigens PD1 and CTLA4 were all higher on infected cells than they were on bystander or UI cells, consistent with infected cells being in an activated state. The homing receptors CD49d, CD29, and CCR6 were more highly expressed on infected than UI or bystander cells both *in vitro* and *in vivo*. However, the homing receptor CXCR5 was not and even showed reduced expression on infected cells *in vitro*. Conversely, the interleukin-7 (IL-7) receptor CD127 was expressed at low levels on infected cells *in vivo* but not *in vitro*. The full set of antigens quantitated on UI, bystander, and infected cells are presented in Figures 1 and S3. Collectively, these results suggest that the phenotypic features of infected and bystander cells are largely shared between *in vivo* and *in vitro* infection conditions, but exceptions exist.

### HIV preferentially targets different subsets of memory CD4^+^ T cells during *in vivo* versus *in vitro* infection

**Figure 2.**
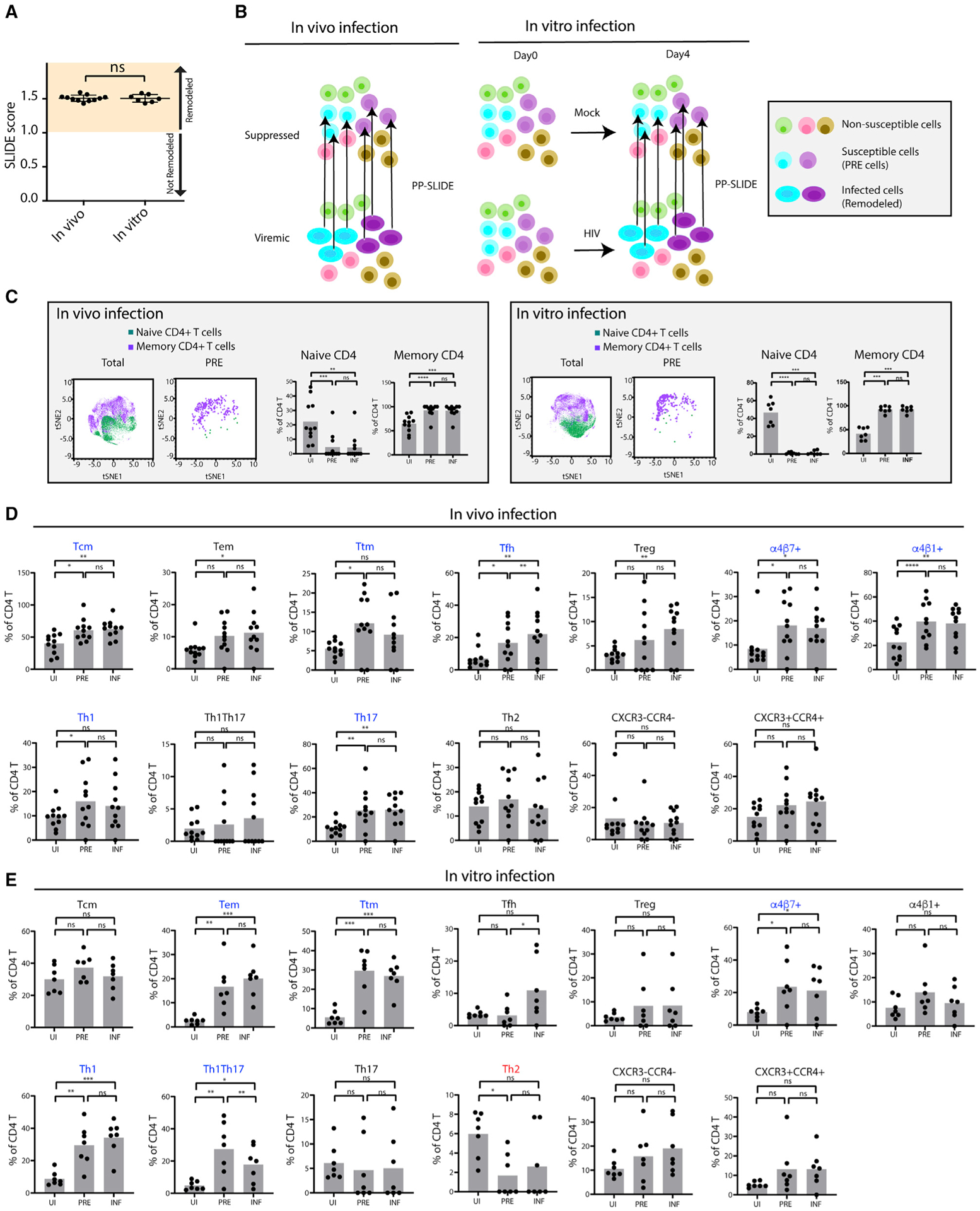
Characterization of *in vivo* and *in vitro* HIV-susceptible cells by PP-SLIDE (A) HIV-infected cells from *in vitro* and *in vivo* specimens are similarly remodeled. Viral-induced remodeling was quantitated by SLIDE, a method that uses k-nearest-neighbor approaches to quantify remodeling based on global changes in expression levels of phenotyping parameters (Sen et al., 2014). A SLIDE score >1.0 indicates remodeling. (B) Schematic of the use of PP-SLIDE to characterize the features of HIV-susceptible cells. For the *in vivo* specimens, we identified, for every HIV-infected cell (represented by the elliptical shapes to signify remodeling) in the viremic specimen, the phenotypically most similar cell in the sample from the same patient at the virally suppressed time point. For the *in vitro* specimens, we identified, for every HIV-infected cell in the HIV-exposed culture, the phenotypically most similar cell in the UI culture. The cells identified by PP-SLIDE are referred to as the predicted precursor cells (PRE cells) and harbor the predicted phenotypes of HIV-susceptible cells before HIV-induced remodeling. In this schematic, the aqua and purple cells are susceptible to HIV infection, whereas the green, pink, and brown ones are not. Comparison of PRE cells to total UI T cells enables assessment of which cellular subsets are preferentially infected by HIV. (C) Memory CD4^+^ T cells are preferential targets of HIV both *in vivo* and *in vitro*. Within each type of specimen, t-distributed stochastic neighbor embedding (tSNE) plots are shown for total CD4^+^ T cells and for the PRE cells of infected cells. Most PRE cells were memory (purple) and not naive (green) cells. Shown are results concatenated from all donors. The same data separated by donor are presented in Figure S4. The bar graphs show the proportions of naive and memory CD4^+^ T cells for all specimens analyzed in this study. ***p < 0.001, ****p < 0.0001 as determined by a Student’s paired t test. (D and E) Frequencies of cellular subsets in UI, PRE, and INF specimens among total CD4^+^ T cells. Subsets were defined as follows: T central memory (Tcm): CD45RO^+^CD45RA^−^CCR7^+^CD27^+^; T effector memory (Tem): CD45RO^+^CD45RA^−^CCR7^−^CD27^−^; T transitional memory (Ttm): CD45RO^+^CD45RA^−^CCR7^−^CD27^+^; T follicular helper (Tfh): CD45RO^+^CD45RA^−^CXCR5^+^PD1^+^; regulatory T cells (Treg): CD45RO^+^CD45RA^−^CD25^+^CD127^−^; the α4β7^+^ subset: CD45RO^+^CD45RA^−^Act1^+^; the α4β1^+^ subset: CD45RO^+^CD45RA^−^CD29^+^CD49d^+^; Th1: CD45RO^+^CD45RA^−^CCR4^−^CXCR3^+^CCR6^−^; Th2: CD45RO^+^CD45RA^−^CCR4^+^CXCR3^−^CCR6^−^; Th17: CD45RO^+^CD45RA^−^CCR4^+^CXCR3^−^CCR6^+^; and Th1/Th17: CD45RO^+^CD45RA^−^CCR4^−^CXCR3^+^CCR6^+^. The CXCR3^−^CCR4^−^ and CXCR3^+^CCR4^+^ populations were pre-gated on CD45RO^+^CD45RA^−^ cells. Gating strategies are shown in Figure S5. Datasets were from the *in vivo* (D) or *in vitro* (E) specimens. *p < 0.05, **p < 0.01, ***p < 0.001, ***p < 0.001 as determined by a Student’s paired t test. ns, not significant. The same datasets comparing PRE and INF cells to UI memory CD4^+^ T cells are presented in Figure S6. Subsets whose frequencies are significantly higher in PRE as compared with UI cells (i.e., those preferentially targeted for infection) are highlighted in blue, whereas those whose frequencies are significantly lower in PRE as compared with UI cells (i.e., those relatively resistant to infection) are highlighted in red. See also Figures S4–S7.

Differentially expressed antigens on HIV-infected cells, relative to UI cells, could reflect preferential infection of cells expressing those markers or result from the up- or downregulation of those markers after HIV infection. For instance, both *in vivo* and *in vitro*, the memory T cell marker CD45RO was expressed at higher levels on infected cells (Figure S3). Although this likely results from HIV preferentially targeting memory CD4^+^ T cells over naive ones, theoretically, it could also result from upregulation of CD45RO on naive cells after infection. Supporting the possibility of HIV-induced remodeling was our finding of significant viral-induced remodeling in infected cells, as determined by SLIDE (Sen et al., 2014), with no difference in the extent of remodeling between the *in vivo* and *in vitro* specimens (Figure 2A). To assess which subsets are preferentially targeted by HIV for infection, we implemented PP-SLIDE (Cavrois et al., 2017; Ma et al., 2020), which takes advantage of the fact that, although HIV infection will change the phenotypes of the cells, some “identity” of the original cell is still retained in a way that can be re-captured by bioinformatics analysis of the high-dimensional datasets. To conduct PP-SLIDE on the *in vivo* specimens, we identified, for every HIV-infected remodeled cell (elliptical-shaped cells in Figure 2B), the phenotypically most similar cell among UI CD4^+^ T cells in the paired, virally suppressed sample. These identified predicted precursor cells, or PRE cells, harbor the predicted phenotypes of the original cells targeted for infection. For the *in vitro* specimens, individual HIV-infected cells from the HIV-exposed culture were mapped to their most-similar cell in the mock-treated culture (Figure 2B). Comparing the PRE cells to the total UI population enabled us to assess which subsets were preferentially targeted for infection, without the confounder of HIV-induced remodeling.

We first determined the frequencies of naive and memory cells among the UI and PRE cells. Although both memory (CD45RO^+^CD45RA^−^) and naive (CD45RO^−^CD45RA^+^) cells were well-represented among total CD4^+^ T cells, PRE cells were almost exclusively of the memory phenotype (Figures 2C and S4). These results suggest that, both *in vivo* and *in vitro*, HIV preferentially targets memory CD4^+^ T cells over naive ones, as opposed to HIV upregulating CD45RO after infection of naive cells. We then assessed other major subsets of T cells among the UI, PRE, and infected CD4^+^ T cells (Figure 2D, 2E, and S5). *In vivo*, Tcm, T transitional memory (Ttm), T follicular helper (Tfh), T helper 1 (Th1), Th17, and cell subsets expressing high levels of the homing receptors α4β7 or α4β1 were preferentially targeted for infection, as defined by significantly higher frequencies among the PRE relative, to the UI, cells. *In vitro*, Tem, Ttm, Th1, Th1/Th17, and cell subsets expressing high levels of α4β7 were preferentially targeted. As all these subsets belong in the memory compartment, we considered the possibility that their observed preferential infection may in large part be due to preferential infection of memory over naive cells (Figure 2C). To test that possibility, we re-analyzed the data using memory CD4^+^ T cells, instead of total CD4^+^ T cells, as the UI control population. Under these conditions, the preferentially targeted subsets for *in vivo* infection were now limited to only Th17 cells and those expressing high levels of α4β1 (Figure S6). This suggests that, with the exception of those two subsets, most subsets observed to be preferentially infected by HIV *in vivo* could be explained by preferential infection of memory over naive cells. We did not observe phenotypic differences in UI CD4^+^ T cells, UI memory CD4^+^ T cells, PRE cells, or HIV-infected cells dependent on whether the ART-suppressed time point was obtained before, versus after, the viremic time point (Figure S7). For the *in vitro* specimens, Tem, Ttm, Th1, and Th1/Th17 cells were found to be at higher levels in PRE cells, relative to memory CD4^+^ T cells, suggesting their preferential susceptibility to HIV infection (Figure S6). Interestingly, although Tem and Ttm were preferentially targeted, Tcm was preferentially spared. These findings are consistent with prior reports of blood-derived Tem being more susceptible than their Tcm counterparts to *in vitro* infection with HIV (Groot et al., 2006; Ma et al., 2020).

Together, these data suggest that, *in vivo*, memory CD4^+^ T cells, particularly Th17 cells and those expressing high levels of α4β1, are preferentially targeted for productive HIV infection. *In vitro*, memory CD4^+^ T cells are also preferentially targeted, but these targeted cells are of the Tem, Ttm, Th1, and Th1/Th17 subsets. A summary of subset features preferentially targeted for infection, or spared from it, are presented in Table S1.

### Identification of antigens remodeled during HIV infection

**Figure 3.**
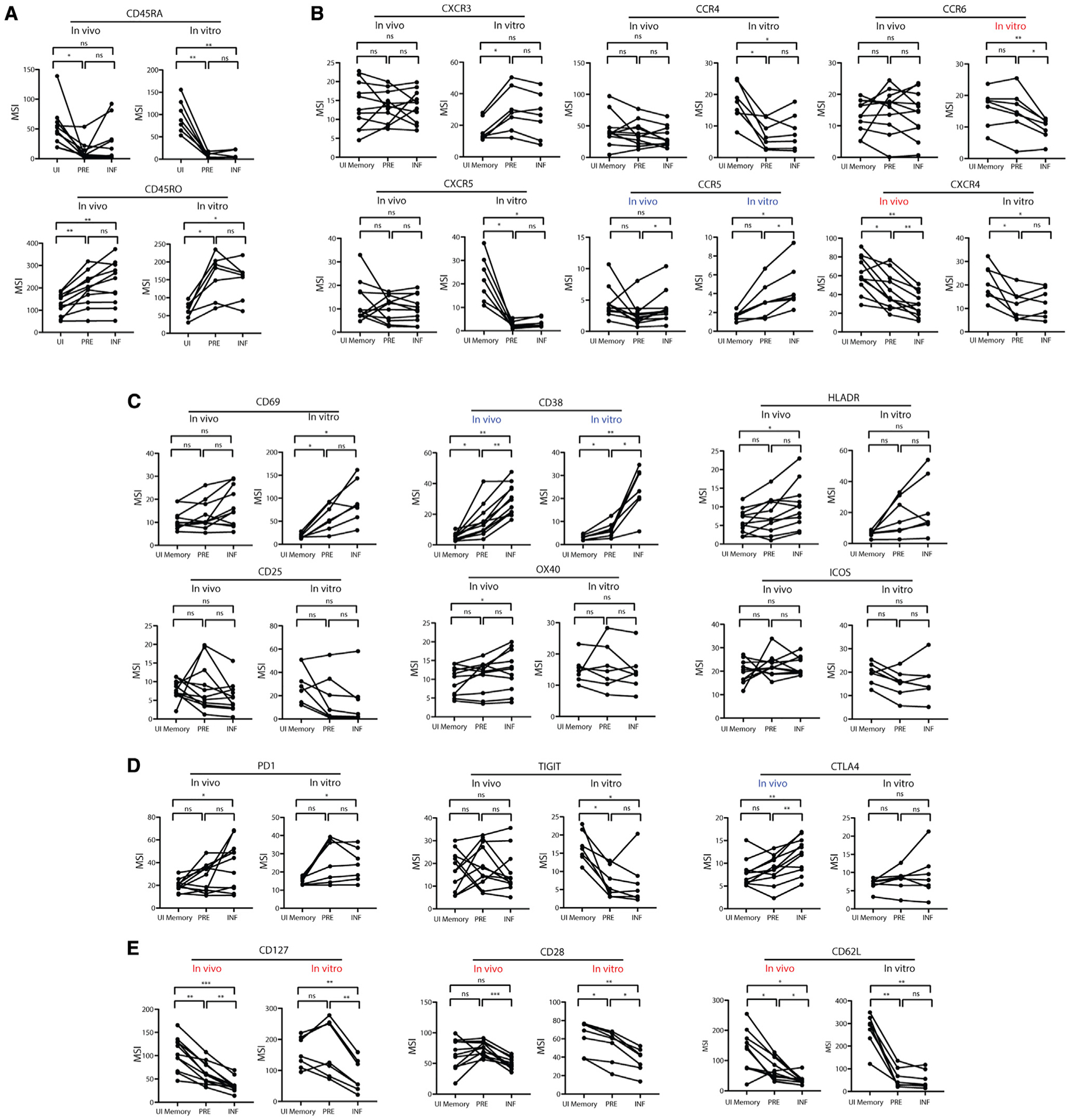
HIV remodels chemokine and cytokine receptors, activation markers, and other surface antigens *in vivo* and *in vitro* (A) The MSIs of the CD45RA and CD45RO were assessed on UI CD4^+^ T cells (UI), PRE cells, and INF cells from the *in vivo* and *in vitro* specimens. The lack of significant differences in CD45RA and CD45RO expression levels in PRE and INF samples indicates HIV does not alter expression levels of these two receptors. (B–E) The MSIs of the indicated chemokine receptors (B), activation markers (C), activation/exhaustion markers (D), and other receptors (E) were assessed on UI memory CD4^+^ T cells (UI memory), PRE cells, and INF cells from the *in vivo* and *in vitro* specimens. *p < 0.05, **p < 0.01, ***p < 0.001 as determined by a Student’s paired t test and adjusted for multiple testing using the Benjamini-Hochberg for FDR. ns, not significant. Remodeled receptors whose MSIs were significantly higher in INF versus PRE cells are highlighted in blue, whereas those whose MSIs were significantly lower in INF as compared with PRE cells are highlighted in red. See also Figure S8.

We next determined which proteins were remodeled by HIV by identifying proteins differentially expressed on HIV-infected cells, relative to the PRE cells. We first confirmed that the memory markers CD45RO and CD45RA were not among those remodeled by HIV, by demonstrating that the mean signal intensity (MSI) of CD45RA and CD45RO were not significantly different between infected and PRE cells (Figure 3A).

We then conducted similar analyses for all the other antigens in our phenotyping panel. We also included UI memory CD4^+^ T cells (taken from the virally suppressed time point for the *in vivo* specimens and the mock-treated sample from the *in vitro* specimens) in our analysis. This enabled the simultaneous identification of antigens marking preferentially infected cells (by comparing PRE and UI memory CD4^+^ T cells) and of antigens remodeled by infection (by comparing the PRE and infected cells). The reason for comparing PRE cells to UI memory CD4^+^ T cells, as opposed to total CD4^+^ T cells, was to avoid the confounding effect of naive cells, which are present in total CD4^+^ T cells but not in PRE cells.

Assessment of the six chemokine receptors in our panel revealed only CCR5 to be consistently upregulated by HIV during infection (Figure 3B). It was only *in vitro*, however, that there was also a trend for pre-selection by HIV for cells expressing high levels of CCR5. Because CCR5 is not only the main HIV co-receptor during transmission but also an activation marker, it is possible that entry of HIV into CCR5-expressing cells activates the cell so as to upregulate CCR5 expression further. Interestingly, the other major HIV-co-receptor, CXCR4, was expressed at lower levels on infected cells relative to their UI counterparts (Figure 3B). *In vivo*, this was due to preferential selection of CXCR4^low^ cells for infection, followed by further downregulation of CXCR4, whereas, *in vitro*, we only observed a selection for CXCR4^low^ cells without a further downregulation of CXCR4. In contrast, CCR6, a marker of Th17 cells, was downregulated by HIV during *in vitro* but not *in vivo* infection (Figure 3B).

We then assessed the remodeling of T cell activation markers. Of the activation markers CD69, CD38, HLA-DR, CD25, Ox40, and inducible T cell co-stimulator (ICOS), only CD38 was upregulated by HIV infection, and this occurred both *in vivo* and *in vitro* (Figure 3C). The high levels of CD38 on HIV-infected cells were due to a combination of both selecting CD38^high^ cells for infection and further CD38 upregulation. The checkpoint molecules PD1, T cell immunoglobulin and ITIM domain (TIGIT), and CTLA4 are preferentially expressed on exhausted T cells but also serve as activation markers. Of these checkpoint molecules, only CTLA4 was upregulated by HIV, and this only occurred *in vivo* (Figure 3D).

Lastly, we assessed whether any proteins were downregulated by HIV both *in vivo* and *in vitro*. CD127, the α-chain of the IL-7 receptor, and CD28, a co-stimulatory receptor targeted for degradation by HIV-1 Nef (Cavrois et al., 2017; Swigut et al., 2001), were downregulated by HIV both *in vivo* and *in vitro*. Additional markers downregulated by HIV included the Tcm markers CD62L and CCR7, although that downregulation only occurred for the *in vivo* specimens (Figures 3E and S8). The remodeling profile of all the antigens not shown in Figure 3 is presented in Figure S8.

In sum, the remodeling analyses revealed a variety of protein antigens that were up- or downregulated by HIV during *in vivo* infection, including receptors for chemokines and cytokines and markers of T cell activation, some of whom could be recapitulated *in vitro*. A summary of protein antigens similarly remodeled in the *in vivo* and *in vitro* specimens are presented in Table S2.

### Markers identified by unbiased clustering enrich for preferential HIV targets among CD4^+^ T cells

**Figure 4.**
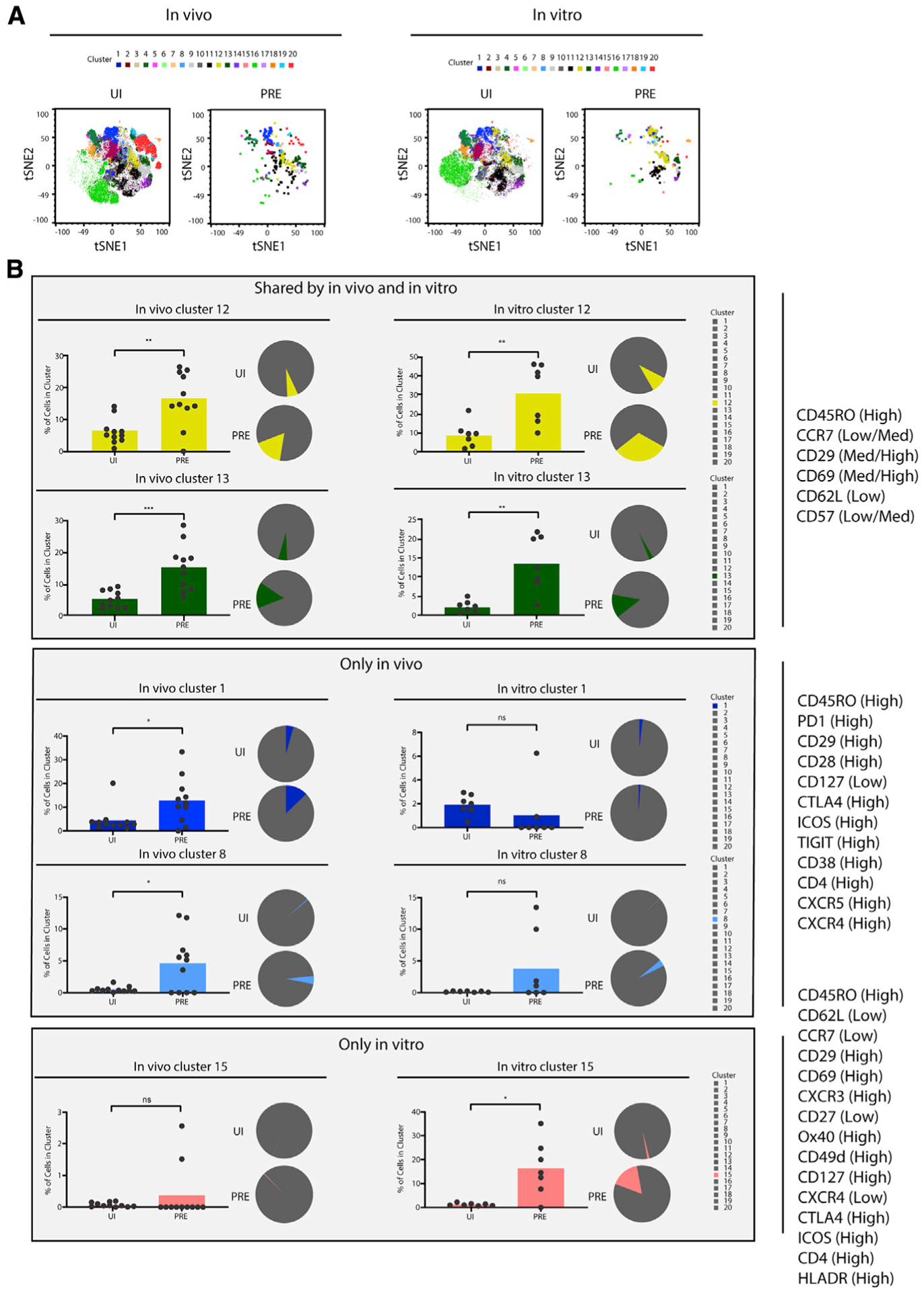
Unbiased clustering identifies clusters of memory CD4^+^ T cells preferentially targeted for HIV infection *in vivo* and *in vitro* (A) tSNE depiction of concatenated data from all the UI CD4^+^ T cells and PRE cells from the *in vivo* (n = 11) and *in vitro* (n = 7) specimens. All *in vivo* and *in vitro* cells were clustered within the same FlowSOM run and colored according to the cluster to which they belonged. The difference in cluster distribution between PRE cells and UI cells suggests a non-random selection of CD4^+^ T cells for infection by HIV. tSNE plots of the same dataset separated by donor specimen are shown in Figure S10. (B) Clusters preferentially targeted for HIV infection both *in vivo* and *in vitro* (clusters 12 and 13), only *in vivo* (clusters 1 and 8), or only *in vitro* (cluster 15) are shown. The proportions of cells that belonged in the indicated cluster, within the UI or PRE populations, are depicted as bar graphs and pie graphs. Clusters that comprise a larger proportion of PRE cells than UI cells are those that are preferentially targeted for infection by HIV. *p < 0.05, **p < 0.01, ***p < 0.001 as determined by a Student’s paired t test. ns, not significant. See also Figures S9–S11.

Having used manual gating and PP-SLIDE to identify subsets of cells that were preferentially targeted for infection and to characterize their remodeling, we then implemented a more unbiased method, FlowSOM, to try to identify novel features of cells preferentially targeted for infection by HIV. FlowSOM divided CD4^+^ T cells from all specimens into 20 clusters (Van Gassen et al., 2015) based on expression levels of CyTOF phenotyping parameters. Some clusters preferentially harbored memory cells, whereas others preferentially harbored naive cells (Figure S9). Consistent with the unequal susceptibility of different cell subsets to HIV infection, PRE cells and the total UI CD4^+^ T cells distributed to different clusters (Figures 4A and S10).

**Figure 5.**
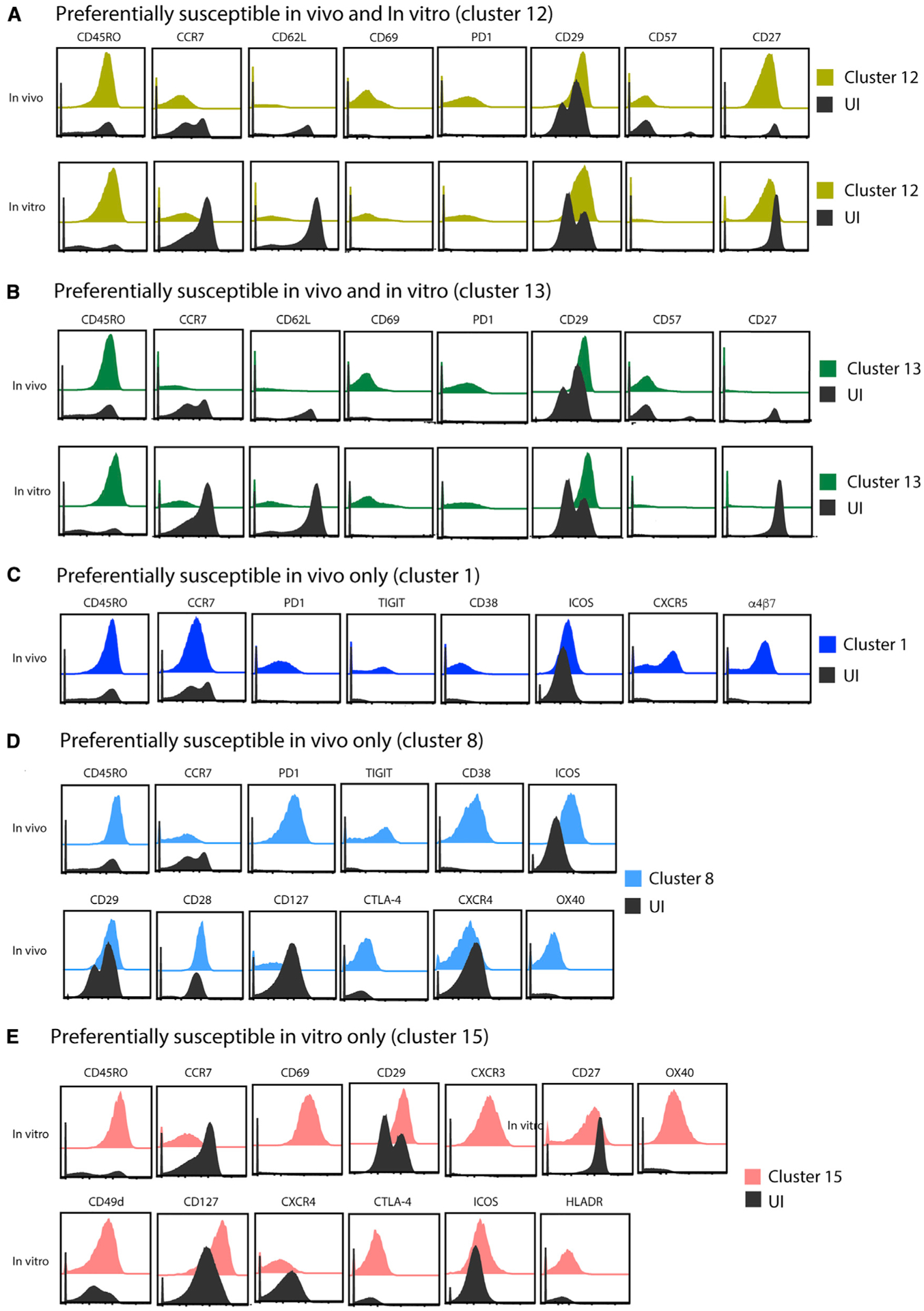
Surface markers differentially expressed on highly susceptible CD4^+^ T cells, related to Figure 4 (A–E) Cell-surface antigens that were most differentially expressed on cells from the preferentially infected clusters identified in Figure 4 are shown. These antigens correspond to those differentially expressed in the two clusters of HIV-susceptible cells both *in vivo* and *in vitro* (A and B), the two clusters of HIV-susceptible cells only *in vivo* (C and D), or the one cluster of HIV-susceptible cells only *in vitro* (E). Select antigens differentially expressed within each cluster, as compared with total UI CD4^+^ T cells, are depicted as histogram plots of concatenated data from all *in vivo* or *in vitro* donors. Clusters 12 and 13 depict both *in vivo* and *in vitro* specimens, clusters 1 and 8 depict the *in vivo* specimens only, and cluster 15 depicts the *in vitro* specimens only.

We then looked for clusters that were preferentially enriched in the PRE cells, relative to the total UI CD4^+^ T cells. These clusters correspond to cellular subsets that are preferentially targeted for HIV infection. Both *in vivo* and *in vitro*, clusters 12 and 13 were significantly enriched among the PRE cells (Figure 4B). Relative to the total UI CD4^+^ T cells, both of these clusters expressed high levels of CD45RO and low levels of CCR7 and CD62L, consistent with their harboring a more Tem-like phenotype than a Tcm-like one. They also expressed more CD69, PD1, and the β1 integrin (CD29), consistent with a more-activated phenotype, and expressed less CD57, consistent with a less terminally differentiated state (Figures 5A and 5B). Although CD27 was expressed at high levels in cluster 12, it was expressed at low levels in cluster 13. Interestingly, we also identified some clusters that were preferentially targeted for infection only *in vivo* or *in vitro*. Clusters 1 and 8 were significantly over-represented in PRE cells *in vivo*, but not *in vitro*, whereas cluster 15 was over-represented in PRE cells *in vitro* but not *in vivo* (Figure 4B). All three of these clusters were memory (CD45RO^high^) and Tem-like (CCR7^low^) but varied in their expression of other antigens (Figures 5C–5E). Importantly, although all the HIV-susceptible clusters that were identified were memory cells, not all clusters of memory cells were preferentially susceptible. For example, clusters 2, 4, and 9 were predominantly of the memory phenotype but were not preferentially targeted by HIV for infection (Figure S9). These preferentially spared clusters differentially expressed some markers, including elevated levels of Tcm markers (CCR7, CD62L, and CD27), relative to the preferentially targeted clusters 12 and 13 (Figure S11).

**Figure 6.**
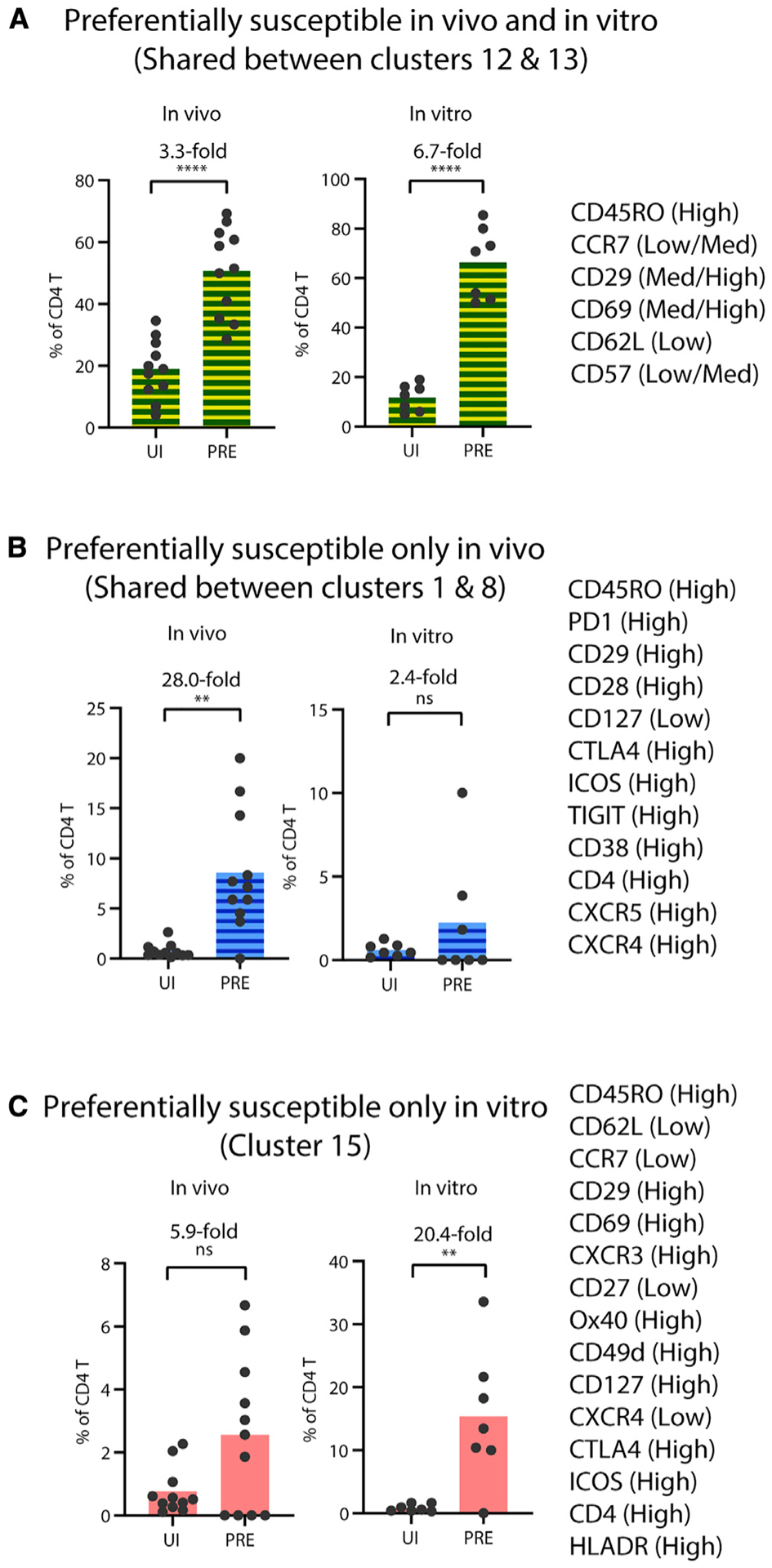
Surface markers differentially expressed on highly susceptible CD4^+^ T cells (A–C) Cell-surface antigens differentially expressed on the indicated cluster(s), relative to total CD4^+^ T cells, were selected for manual gating analyses. These clusters were preferentially targeted in both *in vivo* and *in vitro* HIV infection (A), in *in vivo* infections only (B), or in *in vitro* infections only (C). Phenotypic profiles compiled from these antigens were used to gate the CD4^+^ T cells manually. The bar graphs show the proportion of UI and PRE cells expressing the antigen profiles shown on the right. The fold-differences between the proportions of each subset in PRE versus UI cells are also depicted as numbers on top of each graph. **p < 0.01, ****p < 0.0001 as determined by a Student’s paired t test. ns, not significant.

To validate these findings, we manually searched for a limited set of antigens that were differentially expressed on preferentially infected cells both *in vivo* and *in vitro* (antigen patterns shared by clusters 12 and 13), only *in vivo* (antigen patterns shared by clusters 1 and 8), or only *in vitro* (antigen patterns in cluster 15). Manual gating of the datasets based on the six markers that together define clusters 12 and 13 (CD45RO^high^CCR7^low/med^CD62L^low^CD69^med/high^CD29^med/high^ CD57^low/med^) was sufficient to significantly enrich for PRE cells both *in vivo* and *in vitro* (Figure 6A). Similar analyses using markers shared between clusters 1 and 8 enabled significant enrichment of HIV-susceptible cells *in vivo* (28-fold), but not *in vitro* (Figure 6B). Cluster 15 features were similarly validated by confirming their ability to significantly enrich for *in-vitro*- but not *in-vivo*-susceptible cells (Figure 6C).

**Figure 7.**
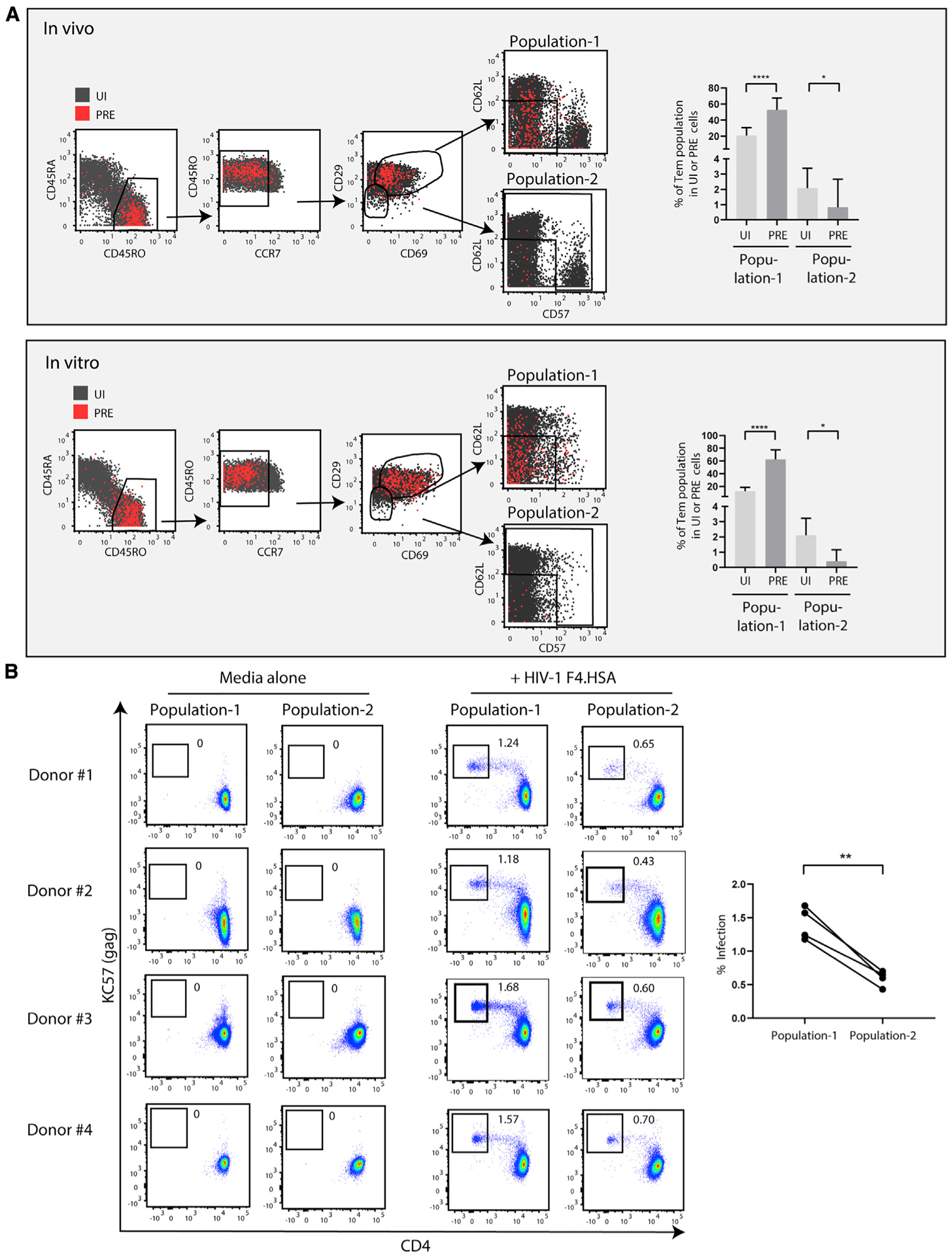
A subset of Tem-like cells sorted based on surface markers defining clusters 12 and 13 are highly susceptible to HIV infection (A) Shown are the CyTOF datasets, with UI CD4^+^ T cells shown in gray and the HIV-susceptible PRE cells shown in red. Cells were pre-gated on live, singlet CD3^+^CD19^−^CD8^−^CD4^+^ T cells. A sequential gating strategy was then implemented using surface markers characteristic of HIV-susceptible cells as defined by clusters 12 and 13. This strategy was used to characterize a final population of “population-1” cells (CD3^+^CD4^+^CD45RO^+^CD45RA^−^CCR7^low/med^CD29^med/high^CD69^med/high^ CD62L^low^CD57^low/med^), which were more abundant among PRE cells than among UI cells. For comparison, we characterized a “population-2” (CD3^+^CD4^+^CD45RO^+^CD45RA^−^CCR7^low/med^CD29^low^CD69^low^ and not CD62L^low^CD57^low/med^) predicted to be much less susceptible to infection because it comprised a significantly lower proportion of PRE cells. The gating strategies are shown on the left, whereas the graphs on the right depict the frequencies of the population-1 and population-2 subsets within the UI and PRE cell populations. Note that the over-representation of population-1 cells among PRE cells suggest their preferential susceptibility to infection, whereas the under-representation of population-2 cells among PRE cells suggest their relative resistance to infection. *p < 0.05, ****p < 0.0001 as determined by a Student’s paired t test. Error bars correspond to the standard deviation. (B) The prediction by PP-SLIDE that the enriched population is more susceptible to HIV infection than is population-2 was validated *in vitro* by sorting those two populations (Figure S12) from PBMCs of four UI donors and then exposing the cells for 4 days to media alone or to the HIV reporter F4.HSA. In all four donors, infection rates were higher in population-1 than they were in population-2. The infection data from all four donors are compiled on the graph on the right. **p < 0.01 as determined by a Student’s paired t test. See also Figure S12.

Finally, we set out to experimentally validate the clustering data. Markers of HIV-susceptible cells that are shared between *in vivo* and *in vitro* specimens would serve as useful tools for establishing *in vitro* models of infection that better mimic HIV-infected cells *in vivo*. Therefore, we set out to confirm that surface antigens identified from clusters 12 and 13 enrich for HIV-susceptible cells. Of the six differentially expressed antigens collectively defining clusters 12 and 13, CD45RO and CCR7 are commonly used to define Tem cells, which are commonly defined as CD45RO^high^ T cells expressing low levels of CCR7. We therefore asked whether Tem cells included cells with differential susceptibilities to *in vitro* infection by HIV. We first gated on CD45RO^high^ CCR7^low/med^ cells and, within that population, determined a gating strategy leading to cells characteristic of clusters 12 and 13 and to cells outside these clusters. The former population we called “population-1” (CD3^+^CD4^+^CD45RO^+^CD45RA^−^CCR7^low/med^CD29^med/high^CD69^med/high^CD62L^low^CD57^low/med^), and the latter “population-2” (CD3^+^CD4^+^CD45RO^+^CD45RA^−^CCR7^low/med^CD29^low^CD69^low^ and not CD62L^low^CD57^low/med^). Although population-1 was predicted to be preferentially susceptible to infection because of their over-representation in PRE relative to UI cells, population-2 was predicted to be relatively resistant because of their under-representation in PRE relative to UI cells (Figure 7A). Population-1 and population-2 cells were sorted from four UI donors (Figure S12) and then exposed to the HIV-1 CCR5-tropic reporter virus F4.HSA. Four days later, infection rates were determined by flow cytometry. In all four donors, population-1 cells were infected at higher rates than the population-2 cells were (Figure 7B), demonstrating that a handful of surface markers defining clusters 12 and 13 could enrich for HIV-susceptible cells. Because clusters 12 and 13 were also preferentially targeted for HIV infection *in vivo*, future studies characterizing *in vitro* HIV infection of these cells may serve as a viable model to better understand active HIV replication in the blood of people living with HIV.

## DISCUSSION

In this study, we take advantage of high-parameter single-cell phenotyping to establish an in-depth view of the features of HIV-infected cells in the blood of individuals with viremia and directly compare them to PBMCs infected *in vitro* with HIV. Using bioinformatics approaches, we assess which cellular subsets are preferentially targeted for infection and which antigens are remodeled by HIV. As resources for the research community, we provide (1) expression levels of each CyTOF phenotyping parameter on UI, infected, and bystander cells; (2) expression levels of each CyTOF phenotyping parameter on PRE cells compared with infected and UI cells, enabling assessment of which antigens are likely to be up- or downregulated by HIV during infection; (3) the proportion of common cellular subsets among UI, PRE, and infected cells, enabling assessment of which subsets are preferentially targeted for productive infection by HIV; and (4) the raw, high-dimensional datasets.

Most prior studies examining the susceptibility of cellular subsets to HIV infection have used *in vitro* systems because direct phenotyping of infected cells from individuals with viremia is more challenging. HIV-infected cells in the blood of individuals are rare relative to frequencies that can be achieved *in vitro*, thereby necessitating approaches that help distinguish true infected cells from background. Similar to a previous study (Pardons et al., 2019), we used two sets of anti-Gag antibodies to increase the signal-to-noise ratio from *in-vivo*-infected cells; however, we used CyTOF, instead of flow cytometry, for our readout. Almost all infected cells from individuals with viremia had downregulated cell-surface CD4, in contrast to *in-vitro*-infected cells, in which only a fraction of the cells had done so. The downregulation of cell-surface CD4 can be mediated by Nef, Vpu, and Env (Doms and Trono, 2000; Lama, 2003; Piguet et al., 1999). Why CD4 downregulation is more potent *in vivo* than *in vitro* is unclear, but could potentially be driven by faster viral replication kinetics *in vivo*, because Vpu and Env are produced relatively late in the HIV replication cycle. Interestingly, the expression pattern of the CCR5 co-receptor was also different, depending on whether the cells were from the *in vivo* or *in vitro* specimens. Although *in vivo* CCR5 levels were equivalent between UI and infected cells, they were higher on *in-vitro*-infected cells. Because CCR5 is a gut-homing chemokine receptor, it is possible that infected cells expressing high levels of CCR5 are rapidly recruited to the gastrointestinal tract, rendering them no longer detectable in the blood.

One main advantage of high-dimensional phenotyping by CyTOF is that it enabled assessment of whether antigens differentially expressed on infected cells likely resulted from preferential infection of cellular subsets differentially expressing those antigens, or HIV-induced changes in antigen expression, or a combination of the two. This distinction was accomplished by PP-SLIDE, a bioinformatics approach, whereby HIV-remodeled cells are traced to their likely original state by matching them to their “nearest-neighbor” cell in an atlas of UI CD4^+^ T cells from the same donor. The approach assumes that, despite HIV-induced remodeling, some of the original “identity” of the infected cell is retained in a manner that can be captured by single-cell, high-dimensional phenotyping, such as that offered by CyTOF. We have previously implemented PP-SLIDE on tonsillar and genital T cells infected *in vitro* with HIV and to trace *ex-vivo*-reactivated reservoir cells to their original, latent state (Cavrois et al., 2017; Ma et al., 2020; Neidleman et al., 2020). We validated the predictions made by PP-SLIDE in various ways. For example, PP-SLIDE predictions that CD127-expressing memory CD4^+^ T cells from tonsils were preferentially spared from productive infection was validated by infecting pre-purified CD127^−^ versus CD127^+^ memory CD4^+^ T cells from the tonsils and demonstrating that CD127^+^ cells preferentially underwent latent infection by HIV (Cavrois et al., 2017; Hsiao et al., 2020). We also demonstrated that surface markers identified by PP-SLIDE on unstimulated reservoir cells enrich for genome-intact and replication-competent latent cells from virally suppressed individuals living with HIV (Neidleman et al., 2020).

In this study, by identifying a set of PRE cells harboring the predicted phenotypes of the cells most susceptible to infection before HIV-induced remodeling, we assessed which antigens were likely up- or downregulated during *in vivo* or *in vitro* infection. Of six canonical activation markers (CD69, CD38, HLA-DR, CD25, Ox40, and ICOS), only CD38 was significantly upregulated on infected cells relative to UI memory CD4^+^ T cells both *in vivo* and *in vitro*. Interestingly, PP-SLIDE suggested that both *in vivo* and *in vitro*, this was due to a combination of HIV preferentially infecting CD38^high^ cells, followed by further upregulation of this activation marker. These results suggest that the well-accepted notion that HIV-infected cells are activated is context specific and depends on the activation markers used to define these cells. In this context, CD38 may be a good universal marker for defining the activated state of HIV-infected cells.

We also identified some surface antigens that, based on PP-SLIDE, were predicted to be downregulated by HIV infection. CD28, a co-receptor that mediates “signal 2” of T cell receptor (TCR) signaling, and CD127, a receptor important in IL-7 signaling, were both expressed at significantly lower levels on infected cells than they were on PRE cells. CD28 downregulation by HIV has been well characterized *in vitro* and demonstrated to be mediated by the Nef protein (Cavrois et al., 2017; Swigut et al., 2001). Our data from the individuals with viremia suggest that Nef-mediated CD28 downregulation also occurs *in vivo*. In contrast, CD127 downregulation by HIV has not previously been reported. In fact, the paucity of CD127-expressing HIV-infected tonsillar T cells was not ascribed to CD127 downregulation but to the inability of CD127-expressing cells to sustain a productive HIV infection (Cavrois et al., 2017; Hsiao et al., 2020). The reason for the discrepancy between blood and tonsil T cells is unclear but may be due to different signaling pathways involving CD127 in these two compartments. Supporting this notion is the observation that CD57, a marker of permissive tonsillar CD4^+^ T cells, is co-expressed with CD127 on CD4^+^ T cells from blood but not from tonsils (Cavrois et al., 2017).

We also used PP-SLIDE to assess which cellular subsets were preferentially targeted for HIV infection *in vivo* and *in vitro*. Because we had access to paired viremic and virally suppressed specimens from the same donors, we compared HIV-infected cells to UI cells from the suppressed time point and not UI cells from the viremic time point because these cells would exhibit bystander effects from the inflammatory environment of active HIV replication.

Numerous subsets were over-represented among PRE cells, relative to UI CD4^+^ T cells (Tcm, Ttm, Tfh, Th1, Th17, and CD4^+^ T cells that were α4β7^+^ or α4β1^+^ for *in vivo* specimens; and Tem, Ttm, Th1, Th1/Th17, and CD4^+^ T cells that were α4β7^+^ for *in vitro* specimens), suggesting preferential infection of these subsets. Because PRE cells consisted almost exclusively of memory T cells, the most useful comparison was between PRE cells and the memory T cells of UI specimens. This comparison revealed that only Th17 cells and memory α4β1^+^ CD4^+^ T cells were preferentially infected *in vivo* and, *in vitro*, Tem, Ttm, Th1, and Th1/Th17 cells. The preferential infection of Th17 cells *in vivo* is consistent with cells expressing the Th17 marker CCR6 harboring more HIV DNA than those lacking expression of this marker in individuals with viremia (Gosselin et al., 2010). The analysis comparing PRE to memory CD4^+^ T cells also revealed memory subsets that were preferentially spared from infection, including CXCR3^−^CCR4^−^cells *in vivo* and Tcm, Tfh, Th17, Th2, and CXCR3^−^CCR4^−^cells *in vitro*. The molecular mechanisms underlying the discrepancies observed between the *in vivo* and *in vitro* data remain to be worked out but may be due to differences between primary isolates and reporter viruses, different kinetics of infection, migration of cell populations into and out of blood during untreated *in vivo* infection, and/or the differences in cell death of cellular subsets in the two systems.

Interestingly, both *in vivo* and *in vitro*, the frequencies of Tfh were significantly higher among the infected cells than they were in PRE cells. Of all the subsets we examined, Tfh were the only ones that exhibited that feature. The higher frequencies of Tfh in infected relative to PRE cells suggest that after infection, HIV may increase co-expression of PD1 and CXCR5, the markers we had used to define Tfh. Therefore, our observed higher frequencies of Tfh in infected cells, relative to UI ones, also reported by others (Baxter et al., 2016; Pardons et al., 2019), may be due not to a preferential infection of Tfh, but rather, the ability of HIV to remodel cells to resemble Tfh. Our *in vitro* data, in fact, suggest that, relative to other memory T cell subsets, Tfh were disfavored for infection, but remodeling of the infected cells made them take on features of Tfh. This observation cautions against making assumptions of cellular susceptibilities based solely on the phenotypes of HIV-infected cells and highlights the complexities associated with characterizing virally remodeled cells.

Because our manual gating approach did not identify memory T cell subsets that were preferentially targeted both *in vivo* and *in vitro*, we turned to unbiased computational clustering approaches to try to identify such subsets. Clustering algorithms identify subsets in a more unbiased and comprehensive manner than manual gating based on a small, select number of subset-defining markers. Of the 20 clusters of CD4^+^ T cells that were defined, 17 consisted predominantly of memory cells, two primarily of naive cells, and one a mix of both. As expected, the clusters containing naive cells were not among those preferentially susceptible to infection. Interestingly, many clusters of memory cells were also not preferentially targeted, consistent with the notion that not all memory CD4^+^ T cells are equally susceptible to infection. Although we identified memory T cell clusters that were preferentially targeted for infection only *in vivo* (clusters 1 and 8) or *in vitro* (cluster 15), we focused on the two (clusters 12 and 13) that were preferentially infected both *in vivo* and *in vitro*. To find a limited number of markers that could identify highly susceptible cells both *in vivo* and *in vitro*, we searched for shared markers between clusters 12 and 13 that were differentially expressed relative to total memory CD4^+^ T cells and identified as highly susceptible cells those that were CD45RO^high^CCR7^low^CD69^high^CD29^high^CD62L^low^CD57^low^. Cells that are CD45RO^high^CCR7^low^ are characteristic of Tem cells, and our identification of these features from our clustering analyses is consistent with Tem cells being preferentially susceptible to infection by HIV (Groot et al., 2006; Ma et al., 2020). We postulated, however, that heterogeneity exists among them and that not all Tem cells are highly permissive and, therefore, designed a sorting experiment to purify Tem-like cells (defined as CD45RO^high^ CCR7^low/med^) expressing the additional features of clusters 12 and 13 (CD69^med/high^CD29^med/high^CD62L^low^CD57^low/med^). *In vitro* infection of these sorted cells confirmed our ability to identify HIV-susceptible versus less-susceptible Tem-like cells. Because the markers of clusters 12 and 13 are characteristic of HIV-susceptible cells both *in vivo* and *in vitro*, PBMCs sorted based on those markers could serve as a model for understanding HIV susceptibility in a system that more closely reflects HIV permissiveness *in vivo*.

In summary, we provide as a resource, datasets of surface antigens that are characteristic of UI, infected, bystander, and computationally predicted HIV-susceptible cells *in vivo* and *in vitro* and describe antigens predicted to be remodeled by HIV. We further provide a sort strategy to isolate, from PBMCs, a population of highly permissive CD4^+^ T cells, which can serve as a model for understanding HIV susceptibility *in vivo* because the same subset was preferentially targeted for infection in individuals with viremia. Future studies to better understand cellular susceptibility to HIV infection in the context of *in vivo* HIV transmission should focus on characterizing HIV-infected cells from mucosal tissues. Such studies will likely require the use of animal model systems, such as non-human primates, because mucosal biopsies from untreated, acutely infected individuals are, for the most, part not feasible.

## STAR★METHODS

### RESOURCE AVAILABILITY

#### Lead contact

Further information and requests for resources and reagents should be directed to and will be fulfilled by the lead contact, Nadia Roan (nadia.roan@gladstone.ucsf.edu).

#### Materials availability

This study did not generate new unique reagents.

#### Data and code availability

The raw CyTOF datasets generated from this study are available for download through the public repository Dryad via the following link: https://doi.org/10.7272/Q6SF2TF6.

### EXPERIMENTAL MODEL AND SUBJECT DETAILS

#### Human subjects

Cryopreserved PBMCs from 11 participants in the UCSF SCOPE cohort were analyzed. The SCOPE study is approved by the University of California, San Francisco (IRB # 10-01330), and all participants provided informed consent before participation. The clinical parameters of the participants are listed below. For each participant, two samples were used: one from when the participant was off therapy and viremic, and second from when the participant was ART-suppressed.

**Table:** 

SCOPE ID	Specimen	Age	Gender	ART	Specimen Date	VL Date	VL (Copies/ml)
3173	Suppressed	37	M	3TC, ATV, ENF	9/29/2004	9/29/2004	< 75
3173	Viremic	38		None	1/11/2005	1/11/2005	473447
3029	Suppressed	42	M	ABC, DDI, EFV, NFV	5/28/2003	5/28/2003	< 75
3029	Viremic	43		None	6/1/2004	6/1/2004	60423
1008	Suppressed	39	M	3TC, LPV/r, D4T, NVP	7/22/2002	7/22/2002	< 50
1008	Viremic	40		None	2/11/2003	2/11/2003	83577
2043	Suppressed	35	M	ABC, IDV, RTV, D4T, NVP	3/5/2001	3/5/2001	< 50
2043	Viremic	38		None	10/27/2003	10/13/2003	154778
1134	Suppressed	39	Transgender F	TRU, ATV, RTV	2/21/2007	3/16/2007	< 75
1134	Viremic	37		None	8/4/2005	8/4/2005	63263
1653	Suppressed	30	Male	ATL	11/1/2012	10/31/2012	< 40
1653	Viremic	28		None	2/10/2011	1/25/2011	52858
1718	Suppressed	29	Male	QUAD	8/11/2014	2/24/2014	< 40
1718	Viremic	27		None	4/30/2012	4/11/2012	43463
1102	Suppressed	29	Male	CBV, ATV, RTV	8/30/2004	8/30/2004	< 75
1102	Viremic	27		None	1/22/2003	1/22/2003	43675
1388	Suppressed	34	Male	DRV, RTV, RGV	7/27/2009	7/27/2009	< 40
1388	Viremic	33		None	7/8/2008	7/2/2008	30120
4011	Suppressed	72	Male	3TC, TDF, EFV	10/11/2007	10/11/2007	< 40
4011	Viremic	71		None	1/26/2006	1/13/2006	22400
4012	Suppressed	60	Male	EPZ, ATV	4/6/2006	4/6/2006	< 50
4012	Viremic	58		None	5/13/2004	5/13/2004	7665

Abbreviations: M: Male; F: Female; ABC: Abacavir; CBV: Combovir; DDI: didanosine; EPZ: Epizicom; TDF: Tenofovir disoproxil fumarate; TRU: Truvada; EFV: Efavirenz; NVP: Nevirapine; ATV: Atazanavir; DRV: Darunavir; IDV: Indinavir; LPV: Lopinavir; NFV: Nelfinavir; RTVB: Ritonavir; ATL: Atripla; QUAD: Elvitegravir/Tenofovir/Emtricitabine/Cobicistat; ENF: Enfuvirtide; RGV: Raltgravir; VL: Viral Load

### METHOD DETAILS

#### Virus production

The CCR5-tropic HIV reporter virus F4.HSA (Cavrois et al., 2017) expresses heat stable antigen (HSA) under the HIV-1 LTR promoter, so cell-surface HSA can be used to identify productively infected cells. F4.HSA virions were generated by transfection of 293T cells. A total of 30 μg F4.HSA plasmid was diluted in 2 mL Optimem (GIBCO) and then mixed with 90 μg polyethylenimine HCL (PEI) (Poly-sciences). This mixture was incubated for 15 min at room temperature, and then added into 293T cells in 30 mL DMEM (Corning) supplemented with 10% fetal bovine serum (FBS, from VWR). Transfection was carried out in a T175 flask (Corning), at a time when 293T cells were at ~50% confluency. After 24 hours, media was changed to D10 media, consisting of DMEM supplemented with 10% fetal bovine serum (FBS) and 1% Penicillin-Streptomycin-Glutamine (Thermofisher Scientific). After another 48 hours, supernatant from the 293T cells was collected and filtered through a 0.22 μm filter (Millipore), and then ultracentrifuged at 4°C for 2 hours at a speed of 20,000 rpm on a Beckman Coulter Optima XE-90. The virus pellet was then resuspended in RPMI (Corning), and viral titer was quantitated using the Lenti-X P24^Gag^ Rapid Titer Kit (Takara).

#### Preparation of blood specimens

Cells from the SCOPE participants were thawed and cultured overnight to allow for antigen expression recovery. To prevent *de novo* infection and cell death during the overnight culture, cells were cultured in the presence of an ART cocktail (50 nM Raltegravir and 0.5 μg/ml T-20, both from NIH AIDS reagent program) and 10 μm of the pan-caspase inhibitor Z-VAD-FMK (R&D Systems Inc). Cells were then prepared for CyTOF analysis as described further below. To generate *in vitro*-infected PBMCs, fresh blood was obtained from reduction chambers (Vitalant Research Institute). For every 10 mL blood, 20 mL FACs buffer (PBS with 2% FBS and 2 mM EDTA) was added. To each 30 blood/FACS buffer mixture, a total of 12.5 mL Ficoll (StemCell Technologies, Inc.) was added by slowly dispensing the Ficoll to the bottom of the tube. The Ficoll-treated cells were then centrifuged at 2,000 rpm at room temperature using an Allegra X-12R (Beckman Coulter) without engaging breaks at the end of the centrifugation. The layer of PBMCs was collected, transferred to a new tube, and washed 3X with FACS buffer. Cell pellets were then resuspended in RP10 media (RPMI supplemented with 10% FBS and 1% Penicillin-Streptomycin-Glutamine). Cells were added to a 96-well U-bottom plate (Falcon) at a concentration of 10^6^ cells/well, with each well containing 200 μL media. Cells were then either left in media alone, or exposed to 200–400 ng p24^Gag^ / well for 4 days. Cells were then collected and cryopreserved to match the *in vivo* specimens which had gone through cryopreservation prior to CyTOF analyses. Similar to the *in vivo* specimens, cells were thawed and cultured overnight in the presence of the ART cocktail and Z-VAD-FMK. Cells were then prepared for CyTOF analysis as described below.

#### Mass cytometry (CyTOF)

Staining was conducted similar to previously described methods (Cavrois et al., 2017). A total of 6 × 10^6^ cells / sample of the cells described above were loaded into each well of a 96-well deep well plate (ThermoFisher Scientific). In addition, as a quality control to validate antibody staining and to confirm lack of variability between sample runs, a batch of uninfected and HIV-infected tonsils generated as described (Cavrois et al., 2017; Ma et al., 2020) was processed through the same protocol in parallel. Of note, paired specimens (from the same HIV+ participant, or *in vitro* uninfected and HIV-infected cultures from the same PBMC donors) were always run within the same batch. Cells were washed twice at 4°C with CyFACs, consisting of 0.1% bovine serum albumin (BSA, Sigma), 0.1% NaAz (Sigma-Aldrich) and PBS (Rockland). Cells were then blocked at 4°C with 1.5% mouse sera (Thermo Fisher), 1.5% rat sera (Thermo Fisher), and 0.3% human AB sera (Sigma-Aldrich) sera. Cells were then washed twice with CyFACs, and then incubated for 45 min at 4°C with the cocktail of surface antibodies listed below in a volume of 100 μl/well. The cells were then washed 3X with CyFACS, and incubated for 30 min at 4°C with the live/dead discriminator 115In-DOTA maleimide diluted 1:1,000 in PBS. Cells were then washed 2X with CyFACS, and incubated at 4°C overnight with 2% paraformaldehyde (PFA, Electron Microscopy Sciences). Cells from each well were then incubated for 30 min at 4°C with 500 μL of Foxp3 Fix/Permeabilization Buffer (Fisher Scientific). Cells from each well were then washed 2X with Permeabilization Buffer (Fisher Scientific), and blocked for 15 min at 4°C with 15 μL mouse serum (Thermo Fisher) and 15 μL rat serum (Thermo Fisher) diluted with 80 μL Permeabilization Buffer. Cells from each well were then washed with Permeabilization Buffer, and incubated for 45 min at 4°C with a cocktail of intracellular antibodies listed below, diluted in 100 μL Permeabilization Buffer. Cells were then washed with CyFACS, and stained for 20 min at room temperature with 250 nM Cell-ID^™^ Intercalator-IR (Fluidigm) diluted in 2% PFA in PBS. Cells were then washed twice with CyFACS and incubated overnight at 4°C. Prior to sample acquisition, cells were washed once with MaxPar® cell staining buffer (Fluidigm), once with Cell Acquisition Solution (CAS, Fluidigm), and then resuspended in 1X EQ Four Element Calibration Beads (Fluidigm) diluted with CAS. The concentration of cells was adjusted to achieve an acquisition speed of ~300 events / second. Samples were injected using a wide-bore (WB) injector, and data acquired on a Helios-upgraded CyTOF2 instrument (Fluidigm) at the UCSF Parnassus Flow Core Facility.

**Table:** 

Antigen Target	Clone	Elemental Isotope	Vendor
HLADR	TU36	Qdot (112Cd)	Life Technolgies
CD49d(α4)	9F10	141Pr	Fluidigm
CD19	HIB19	142Nd	Fluidigm
CD57	HNK-1	143Nd	In-house
CCR5	NP6G4	144Nd	Fluidigm
CTLA-4*	14D3	145Nd	In-house
CD8	RPA-T8	146Nd	Fluidigm
CD7	CD76B7	147Sm	Fluidigm
ICOS	C398.4A	148Nd	Fluidigm
CCR4	L291H4	149Sm	Fluidigm
Gag*	FH190-1-1	150Nd	In-house
CD103	Ber-ACT8	151Eu	Fluidigm
TCRγδ	11F2	152Sm	Fluidigm
CD62L	DREG56	153Eu	Fluidigm
TIGIT	MBSA43	154Sm	Fluidigm
CCR6	11A9	155Gd	In-house
CD29(β1)	TS2/16	156Gd	Fluidigm
OX40	ACT35	158Gd	Fluidigm
CCR7	G043H7	159Tb	Fluidigm
CD28	CD28.2	160Gd	Fluidigm
CD45RO	UCHL1	161Dy	In-house
CD69	FN50	162Dy	Fluidigm
CXCR3	G025H7	163Dy	Fluidigm
PD-1	EH12.1	164Dy	In-house
CD127	A019D5	165Ho	Fluidigm
CXCR5	RF8B2	166Er	In-house
CD27	L128	167Er	Fluidigm
CD30	BERH8	168Er	In-house
CD45RA	HI100	169Tm	Fluidigm
CD3	UCHT1	170Er	Fluidigm
Gag-Ab2 (mix of antibodies) *	71-31, 91-5, 241-D, AG3.0	171Yb	In-house
CD38	HIT2	172Yb	Fluidigm
α4β7	Act1	173Yb	In-house
CD4	SK3	174Yb	Fluidigm
CXCR4	12G5	175Lu	Fluidigm
CD25	M-A251	176Yb	In-house

*: Intracellular antibodies

#### Sorting

The sorting strategy to isolate Population-1 and Population-2 was designed based on a combination of assessing expression levels of each antigen on PRE versus total memory CD4+ T cells, determining how large were the populations expressing a particular pattern of antigens, and the availability of robust antibodies in an appropriate channel for sorting. This resulted in the design of the panel shown below. Fresh blood was obtained from reduction chambers (Vitalant Research Institute) of uninfected donors. PBMCs were purified by Ficoll as described above. CD4+ T cells were then purified by negative selection (Stem Cell Technologies), and then depleted of naive cells using CD45RA beads (Miltenyi Biotec). The resulting memory CD4+ T cells were stained for 10 min at room temperature with Zombie Aqua (Biolegend), washed once with FACS buffer, and then stained for 30 min at room temperature with the sorting antibodies (see table below), diluted in Brilliant Stain Buffer (BD Biosciences). The cells were then washed twice with FACS buffer, and sorted using an FACSAria^™^ II instrument (BD Biosciences). Of note, the anti-CD3 antibody used for sorting (clone SK7) does not activate T cells during the sorting process (Hsiao et al., 2020; Ma et al., 2020; Neidleman et al., 2020). Sorted cells were cultured in 96 well U bottom plates (Falcon) at a concentration of 0.5 × 10^6^ cells/well, and mock-treated or infected with 60 ng/well of F4.HSA HIV-1 for 4 days. To assess infection levels, cells were collected, stained with Zombie Aqua (biolegend) and the FACS antibodies listed below, and fixed for 30 min at room temperature with 1% PFA. Intracellular staining for p24^Gag^ was then performed by staining at 4°C for 30 min with FITC-labeled KC57 diluted in Permeabilization Buffer (Fisher Scientific). Cells were then washed twice with FACS buffer, and analyzed on an LSRFortessa^™^ flow cytometer (BD Biosciences).

**Table:** 

Vendor	Catalog Number	Clone	Antigen Target
Biolegend* #	344818	SK7	APC/Cyanine7 Mouse Anti-Human CD3
Biolegend* #	317410	OKT4	PE Mouse Anti-Human CD4
Biolegend* #	344742	SK1	Brilliant Violet 605 Mouse Anti-Human CD8
Biolegend* #	304112	HI100	APC Mouse Anti-Human CD45RA
BD biosciences*	564291	UCHL1	BUV395 Mouse Anti-Human CD45RO
Biolegend*	353236	G043H7	PE/Dazzle 594 Mouse Anti-Human CCR7 (CD197)
BD biosciences*	563808	DREG-56	BV650 Mouse Anti-Human CD62L
Invitrogen*	25057742	TB01	PE/Cyanine7 Mouse Anti-Human CD57
BD biosciences*	562884	FN50	BV421 Mouse Anti-Human CD69
Bio-RAD*	MCA2028F	12G10	FITC Mouse Anti-Human CD29
BioLegend* #	423102		Live/Dead-Zombie Aque
Biolegend#	304224	UCHL1	BV421 Mouse Anti-Human CD45RO
Beckman Coulter#	6604665	KC57	FITC Mouse Anti-HIV-1 Core

* Antibodies used for sorting

# Antibodies were used for analytical flow cytometry

#### CyTOF data analysis

CyTOF datasets were normalized to EQ calibration beads to minimize variability in intra-machine performance (including between different runs) and gated within FlowJO software (BD Biosciences) for CD4+ T cells (defined as live, singlet CD3+CD19−CD8−CD4+ cells from the suppressed/uninfected specimens), memory CD4+ T cells (defined as live, singlet CD3+CD19−CD8−CD4+CD45RO+CD45RA−cells from the suppressed/uninfected specimens), bystander cells (defined as CD3+CD19−CD8−KC57−Gag− from the viremic/infected specimens), and productively infected cells (defined as CD3+CD19−CD8−KC57+Gag+CD4− from the viremic/infected specimens). Of note, our method of identifying productively infected cells would not detect infected cells harboring defective provirus incapable of producing Gag protein, nor would they detect latently infected cells not producing Gag protein.

Predicted Precursor (PRE) cells were derived from the productively infected cells similar to recently described methods (Ma et al., 2020; Neidleman et al., 2020). Of note, PP-SLIDE has proven effective as a predictive algorithm even when low numbers of query events (< 100 cells) are used to identification of PRE cells (Neidleman et al., 2020). In this study, the following steps were implemented to identify PRE cells:

1. Data cleanup and standardization:
  CD3+CD19−CD8−CD4+ T cells from the suppressed/uninfected specimens and HIV-infected cells from the viremic/infected specimens were gated out and exported using Flowjo10. The following parameters, which do not contain useful information for identifying the original cell type, were removed from the analysis:

**Table:** 

Non-informative markers	Live/dead staining, event length, beads channel, DNA, time, background channel (190), and other non-cell markersComments
Infection markers	KC57, Gag antibody mixtures
Marker highly modified by HIV infection and not informative for PRE analysis	CD4
Markers used in upstream gating analysis	CD19, CD8
  Raw expression values (signal intensity) of selected markers from each cell in the exported files were transformed by the inverse hyperbolic function (arcsinh) transformation as follows, in order to standardize the range of raw expression level scales:
  $$\text{arsinh}(x)=\text{ln}\left(x+\sqrt{x^{2}+1}\right)$$
2. Identification of PRE cell for each HIV-infected cell:
  The Euclidean distance (*d*_*F_U*_) between each productively-infected cell *F* and each uninfected cell *U* (from the virally-suppressed sample, or the uninfected *in vitro* specimen) was calculated as follows:
  $$d_{F_{-}U}=\sqrt{\sum_{i=1}^{n}{\left(F_{i}-U_{i}\right)}^{2}}$$
  where n is the number of parameters analyzed and *i* refers to the parameter being analyzed. For example, for parameter 1, *F*_*i*_ − *U*_*i*_ would correspond to the value of parameter 1 on the infected cell minus the value of parameter 1 on the uninfected cell. For each infected cell *F*, the *d*_*F_U*_ of all the suppressed/uninfected cells U were sorted from lowest to highest to identify the shortest *d*_*F_U*_ value. This corresponds to the *k = 1* nearest neighbor uninfected cell for that infected cell *F*, or the PRE cell. After identifying the PRE cells corresponding to each infected cell, the expression values corresponding to the original data matrix were exported as a new FCS file for downstream analysis. These PRE cells correspond to a subset of the original data matrix corresponding to total uninfected cells. The FCS files of the PRE cells were further analyzed in FlowJO.

Cell populations (uninfected, infected, bystander, and PRE cells) were analyzed by manual gating in FlowJO. tSNE was used for data visualization, and was performed in Cytobank, with the following settings: Iteration = 16000; Perplexity = 45; Theta = 0.5. Markers used in the gating strategy (CD19, CD8, KC57, Gag) were excluded as tSNE parameters. FlowSOM (Van Gassen et al., 2015) was used to identify clusters from the high-dimensional CyTOF datasets. FlowSOM was performed in Cytobank, using the following settings: hierarchical consensus; metaclusters = 20; clusters = 225, iterations = 10, using the same parameters as those used for tSNE. The contribution of clusters with the uninfected and PRE cell populations were calculated by determining, for each specimen, the percentages of these clusters in these cell populations.

#### Raw Datasets

Raw datasets of CD4+ T cells, memory CD4+ T cells, infected cells, bystander cells, and PRE cells are available in the public repository Dryad, and accessible via the following link: https://doi.org/10.7272/Q6SF2TF6.

### QUANTIFICATION AND STATISTICAL ANALYSIS

SLIDE calculations to quantitate viral-induced remodeling were conducted using methods recently described (Ma et al., 2020; Sen et al., 2014) using the R package SLIDE (Mukherjee et al., 2018). Statistical details of experiments are displayed in the figures and figure legends. Unless otherwise indicated, all statistical analysis in the figures were conducted on all *in vivo* (n = 11 donors) and *in vitro* (n = 7 donors) specimens analyzed in this study. Mean signal intensity (MSI) levels were calculated from the CyTOF datasets in FlowJO and R, and compared between populations using the Student’s two-sided paired t tests. P values were adjusted for multiple testing using False Discovery Rate (FDR) via the Benjamini-Hochberg method. FDR adjusted P values that were less than 0.05 were considered as significant. Error bars correspond to standard deviation (SD).

## Supplementary Material

1

2

## Figures and Tables

**Table:** KEY RESOURCES TABLE

REAGENT or RESOURCE	SOURCE	IDENTIFIER
Antibodies		
HLADR	Thermofisher	Cat#Q22158
CD49d (α4)	Fluidigm	Cat#3141004B
CD19	Fluidigm	Cat#3142001B
CD57	Biolegend	Cat#359602
CCR5	Fluidigm	Cat#3144007A
CTLA-4	Fisher Scientific	Cat#5012919
CD8	Fluidigm	Cat#3146001B
CD7	Fluidigm	Cat#3147006B
ICOS	Fluidigm	Cat#3148019B
CCR4	Fluidigm	Cat#3149029A
KC57	Beckman Coulter	Cat#IMBULK1B
CD103	Fluidigm	Cat#3151011B
TCRgd	Fluidigm	Cat#3152008B
CD62L	Fluidigm	Cat#3153004B
TIGIT	Fludigm	Cat#3154016B
CCR6	BD Biosciences	Cat#559560
CD29(β1)	Fludigm	Cat#3156007B
OX40	Fluidigm	Cat#3158012B
CCR7	Fluidigm	Cat#3159003A
CD28	Fluidigm	Cat#3160003B
CD45RO	Biolegend	Cat#304239
CD69	Fluidigm	Cat#3162001B
CXCR3	Fluidigm	Cat#3163004B
PD-1	BD Biosciences	Cat#562138
CD127	Fluidigm	Cat#3165008B
CXCR5	BD Biosciences	Cat#552032
CD27	Fluidigm	Cat#3167006B
CD30	BD Biosciences	Cat#555827
CD45RA	Fluidigm	Cat#3169008B
CD3	Fluidigm	Cat#3170001B
HIV-1 Gag antibody 71-31	NIH AIDS reagent program	N/A
HIV-1 Gag antibody 91-5	NIH AIDS reagent program	N/A
HIV-1 Gag antibody 241-D	NIH AIDS reagent program	N/A
HIV-1 Gag antibody AG3.0	NIH AIDS reagent program	N/A
CD38	Fluidigm	Cat#3172007B
A487(APC)	Gift from E. Butcher Lab	N/A
CD4	Fluidigm	Cat#3174004B
CXCR4	Fluidigm	Cat#3175001B
CD25	BD Biosciences	Cat#555430
CD3 (APC/Cyanine7)	Biolegend	Cat#344817
CD4 (PE)	Biolegend	Cat#317410
CD8 (Brilliant Violet 605)	Biolegend	Cat#344742
CD45RA (APC)	Biolegend	Cat#304112
CD45RO (BUV395)	BD biosciences	Cat#564291
CCR7 (PE/Dazzle^™^ 594)	Biolegend	Cat#353236
CD62L (BV650)	BD Biosciences	Cat# 563808
CD57 (PE/Cyanine7)	Invitrogen	Cat#25057742
CD69 (BV421)	BD Biosciences	Cat#562884
CD29 (FITC)	Bio-RAD	Cat#MCA2028F
Live/Dead-Zombie Aque	Biolegend	Cat# 423102
CD45RO (BV421)	Biolegend	Cat# 304224
Anti-HIV-1 Core (FITC)	Beckman Coulter	Cat# 6604665
Bacterial and virus strains		
F4-HSA (NL-HSA.6ATRi-C.109FPB4.ecto)	https://pubmed.ncbi.nlm.nih.gov/28746881/	N/A
Biological samples		
PBMCs from people living with HIV (SCOPE cohort)	https://hividgm.ucsf.edu/scope-study	N/A
PBMCs from HIV-uninfected donors	https://www.vitalant.org	N/A
Chemicals, peptides, and recombinant proteins		
Paraformaldehyde	Electron Microscopy Sciences	Cat#15710
Fetal Bovine Serum	VWR	Cat#97068-085
Metal Contaminant-Free PBS	Rockland	Cat#MB-008
Normal Mouse Serum	ThermoFisher	Cat#10410
Normal Rat Serum	ThermoFisher	Cat#10710C
Human Serum From Male AB Plasma	Sigma-Aldrich	Cat#H4522
Intracellular Fixation & Permeabilization Buffer	ThermoFisher	Cat#88-8823-88
Permeabilization Buffer	ThermoFisher	Cat#00-8333-56
Iridium Interchelator Solution	Fluidigm	Cat#201192B
MaxPar® cell staining buffer	Fluidigm	Cat#201068
Cell acquisition solution	Fluidigm	Cat#201240
EQ Four Element Calibration Beads	Fluidigm	Cat#201078
Opti-MEM	GIBCO	Cat#31985062
polyethylenimine HCL (PEI)	Polysciences	Cat#24765
Brilliant Stain Buffer	BD Biosciences	Cat#563794
Z-VAD-FMK	R&D Systems Inc	Cat# FMK001
T-20	NIH AIDS reagent program	N/A
Raltegravir	NIH AIDS reagent program	N/A
Critical commercial assays		
MaxPar® X8 Antibody Labeling Kit	Fluidigm	Cat#201169B
EasySep^™^ Human CD4+ T cell Isolation Kit	STEMCELL	Cat#17952
CD45RA MicroBeads, human	Miltenyi Biotec	Cat#130-045-901
Deposited data		
Raw CyTOF datasets	This paper	https://doi.org/10.7272/Q6SF2TF6
Software and algorithms		
FlowJo	BD Biosciences	https://www.flowjo.com/
Cytobank	Cytobank	https://www.cytobank.org/

## Highlights

- The CD4 T cell subsets most susceptible to HIV infection differ *in vivo* versus *in vitro*
- Multiple chemokine and cytokine receptors are remodeled by HIV *in vivo*
- HIV remodels infected cells *in vivo* to resemble Tfh
- Identification of subset of highly permissive T cells shared *in vivo* and *in vitro*
