# Supplementary material for: Characterization of HIV-induced remodeling reveals differences in infection susceptibility of memory CD4+ T cell subsets in vivo
Source: Cell Rep. Author manuscript; Available in PMC 2021 Jun 14. (PMC8202093; doi:10.1016/j.celrep.2021.109038)
Supplement: 1 [file NIHMS1708597-supplement-1.pdf]

Cell Reports, Volume *35*

Supplemental information

Characterization of HIV-induced remodeling reveals differences in infection susceptibility of memory CD4$^+$ T cell subsets *in vivo*

Guorui Xie, Xiaoyu Luo, Tongcui Ma, Julie Frouard, Jason Neidleman, Rebecca Hoh, Steven G. Deeks, Warner C. Greene, and Nadia R. Roan

SUPPLEMENTARY TABLES

No selection *in vivo* or *in vitro*	Preferentially infected both *in vivo* and *In vitro*	Preferentially infected only *in vivo*	Preferentially infected only *in vitro*	Preferentially spared both *in vivo* and *In vitro*	Preferentially spared only *in vivo*	Preferentially spared only *in vitro*
Treg<br>CXCR3-CCR4-<br>CXCR3+CCR4+	Ttm<br>α4β7<br>Th1	Tcm<br>Tfh<br>α4β1<br>Th17	Tem<br>Th1Th17	None	None	Th2

Table S1. Subsets preferentially targeted for HIV infection *in vivo* and *in vitro* – Related to Figure 2.

No change *in vivo* or *in vitro*	Upregulated both *in vivo* and *In vitro*	Upregulated only *in vivo*	Upregulated only *in vitro*	Downregulated both *in vivo* and *In vitro*	Downregulated only *in vivo*	Downregulated only *in vitro*
CD45RA<br>CD45RO<br>CXCR3<br>CCR4<br>CXCR5<br>CD69<br>HLADR<br>CD25<br>OX40<br>ICOS<br>PD1<br>TIGIT<br>CD29<br>CD103<br>α4β7<br>CD27<br>CD30<br>CD7<br>TCRγδ<br>CD57<br>CD30	CCR5<br>CD38	CTLA4	None	CD127<br>CD28<br>CD4	CXCR4<br>CD62L<br>CCR7	CCR6<br>CD49d

Table S2. Antigens remodeled during HIV infection *in vivo* and *in vitro* – Related to Figure 3.

SUPPLEMENTARY FIGURES

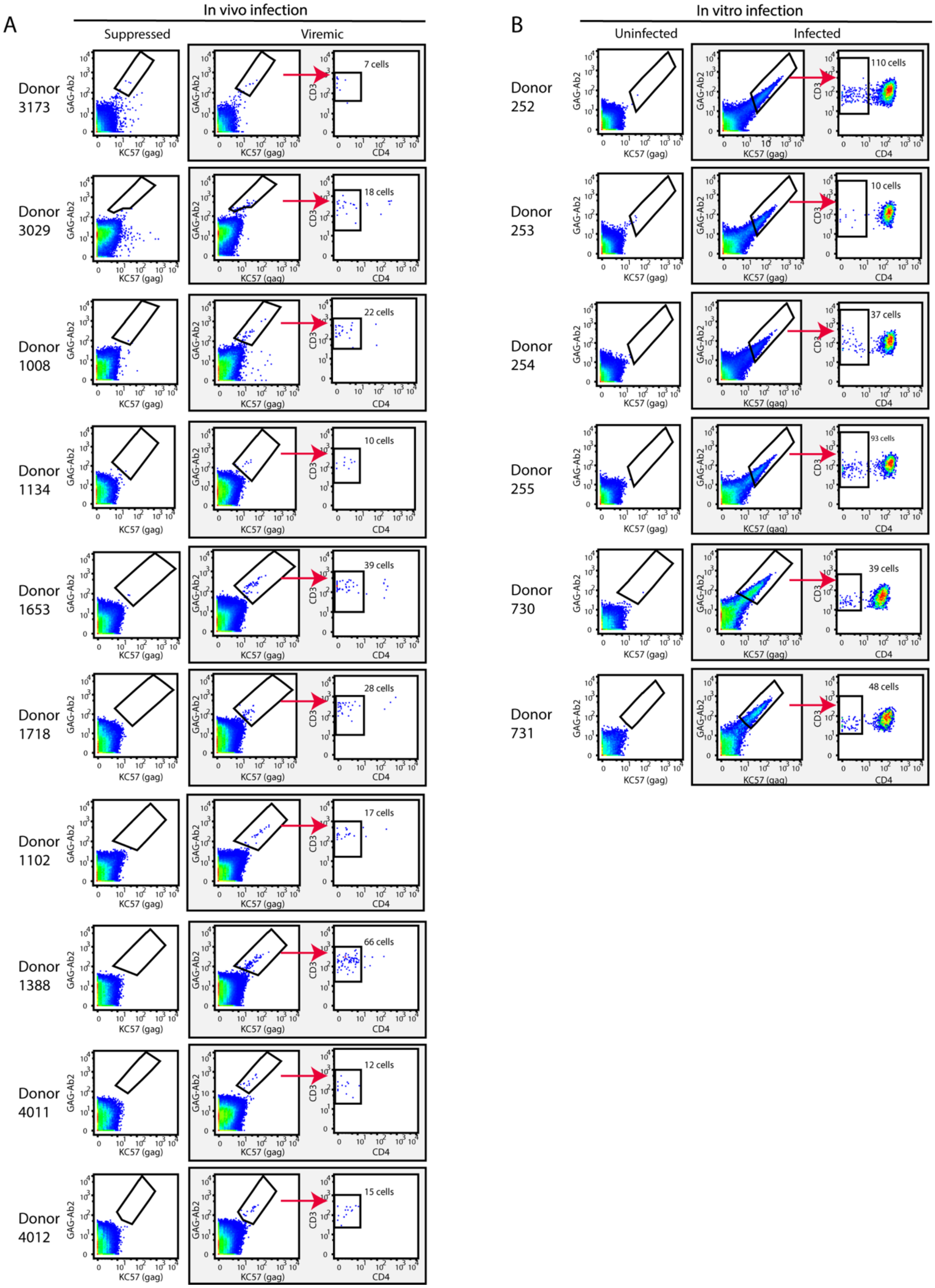

Figure S1. Identification of *in vivo* and *in vitro* HIV-infected cells – Related to Figure 1. HIV-infected T cells were defined as CD3+CD8- cells binding both sets of anti-Gag antibodies (KC57 and GAG-Ab2), and that had downregulated cell-surface CD4. **A)** CD3+CD8- cells from the same individuals at a virally-suppressed timepoint vs. a viremic timepoint, showing CD4-downregulation on infected cells from the viremic timepoint. **B)** CD3+CD8- cells from mock-treated ("Uninfected") vs. HIV-exposed PBMC cultures ("Infected"), showing further gating on CD4-downregulated cells among the Gag-expressing cells. Together with the donors presented in Fig. 1A, a total of 11 *in vivo* specimens and 7 *in vitro* specimens were analyzed. The dual-Gag positive gates were tailored to minimize the numbers of cells in the suppressed/uninfected specimens and maximize the number of cells in the viremic/infected specimens.

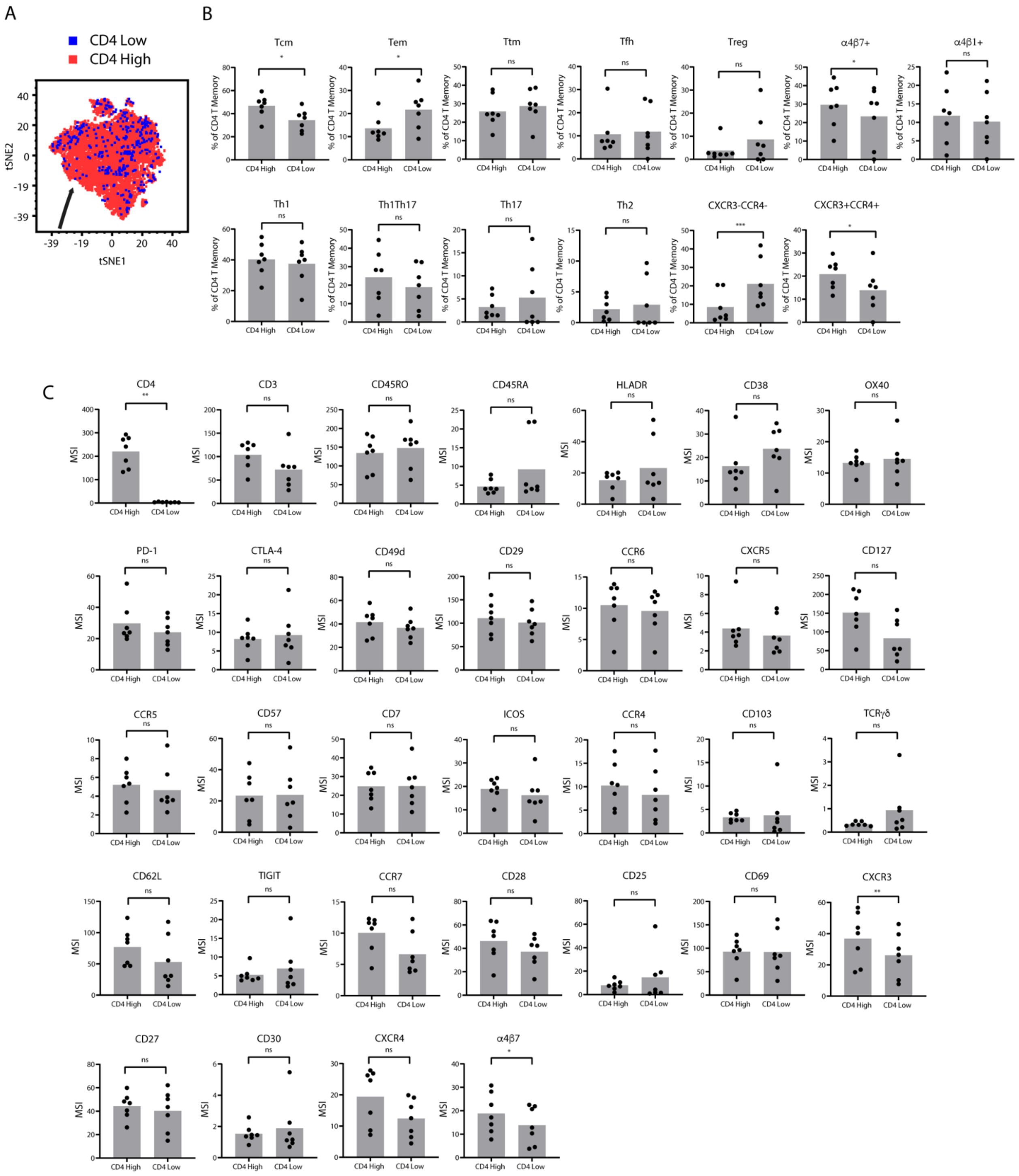

Figure S2. Subset distribution and antigen expression levels among CD4^{high} vs. CD4^{low} HIV-infected cells generated from *in vitro* infection – Related to Figure 1. A) Comparison of the CD4^{high} vs. CD4^{low} HIV-infected (HSA+) cells from the *in vitro* cultures revealed largely

overlapping populations of cells as revealed by tSNE, although a region preferentially devoid of CD4^{low} cells was apparent (arrow). **B)** Comparison of the distribution of the indicated subset among the CD4^{high} and CD4^{low} infected cell populations revealed the CD4^{low} cells to be enriched for Tem and CXCR3-CCR4- cells, and the CD4^{high} cells to be enriched for Tcm, CXCR3+CCR4+, and $\alpha4\beta7$+ cells. * $p < 0.05$ and *** $p < 0.001$ as determined by a Student's paired t-test. **C)** The mean signal intensity (MSI) of the indicated antigen was compared between the CD4^{high} and CD4^{low} infected cell populations. * $p < 0.05$ and ** $p < 0.01$ as determined by a Student's paired t-test and adjusted for multiple testing using the Benjamini-Hochberg for FDR. ns: not significant.

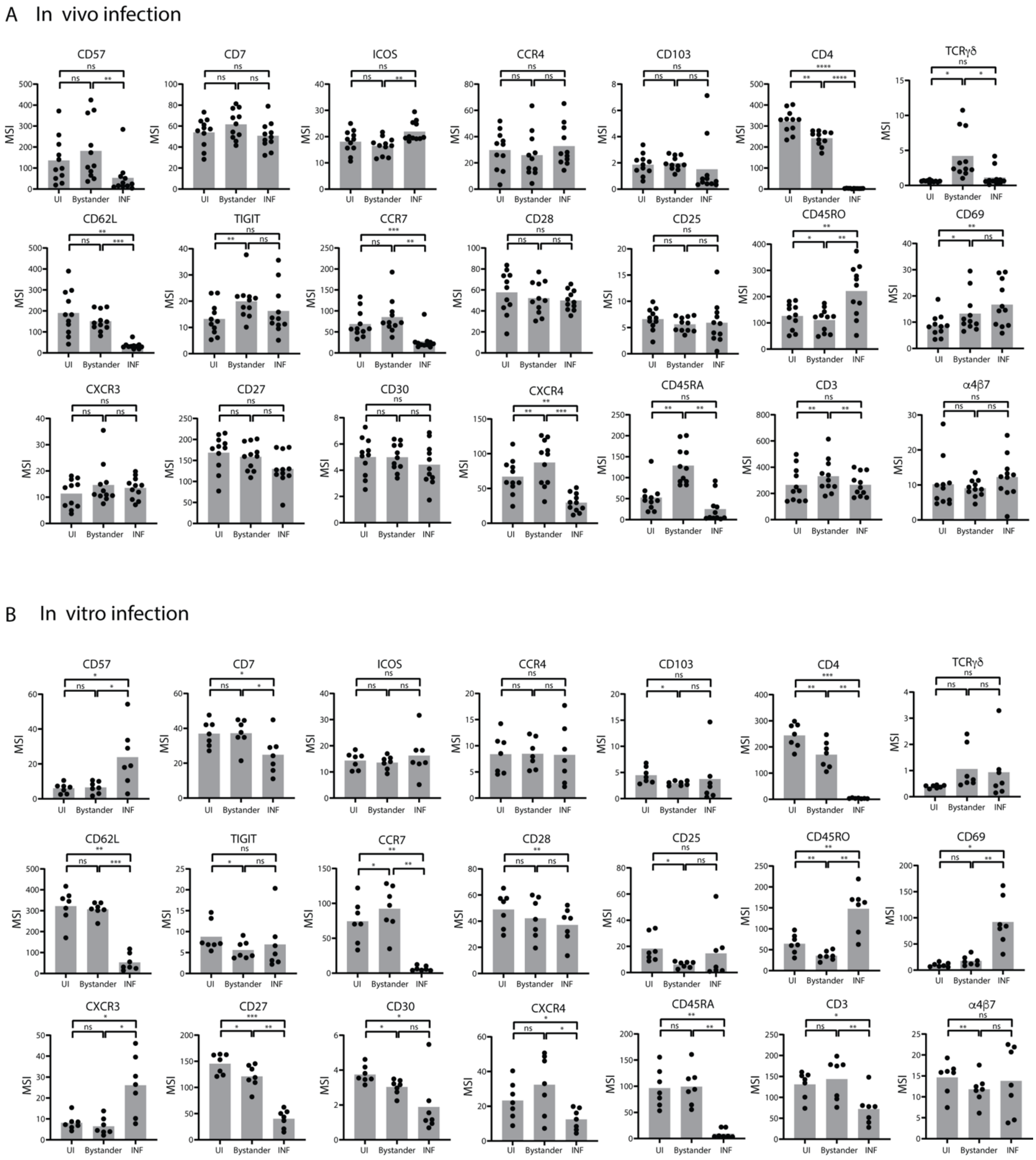

Figure S3. Expression levels of surface and intracellular antigens in uninfected, bystander, and HIV-infected cells *in vivo* and *in vitro* – Related to Figure 1. Shown are the mean signal intensity (MSI) levels of the indicated antigens among uninfected (UI), bystander, and infected (INF) cells, on *in vivo* **(A)** or *in vitro* **(B)** specimens. * $p < 0.05$, ** $p < 0.01$, *** p <

0.001 and **** $p < 0.0001$ as determined by a Student's paired t-test and adjusted for multiple testing using the Benjamini-Hochberg for FDR. ns: not significant. See Fig. 1 for additional antigens.

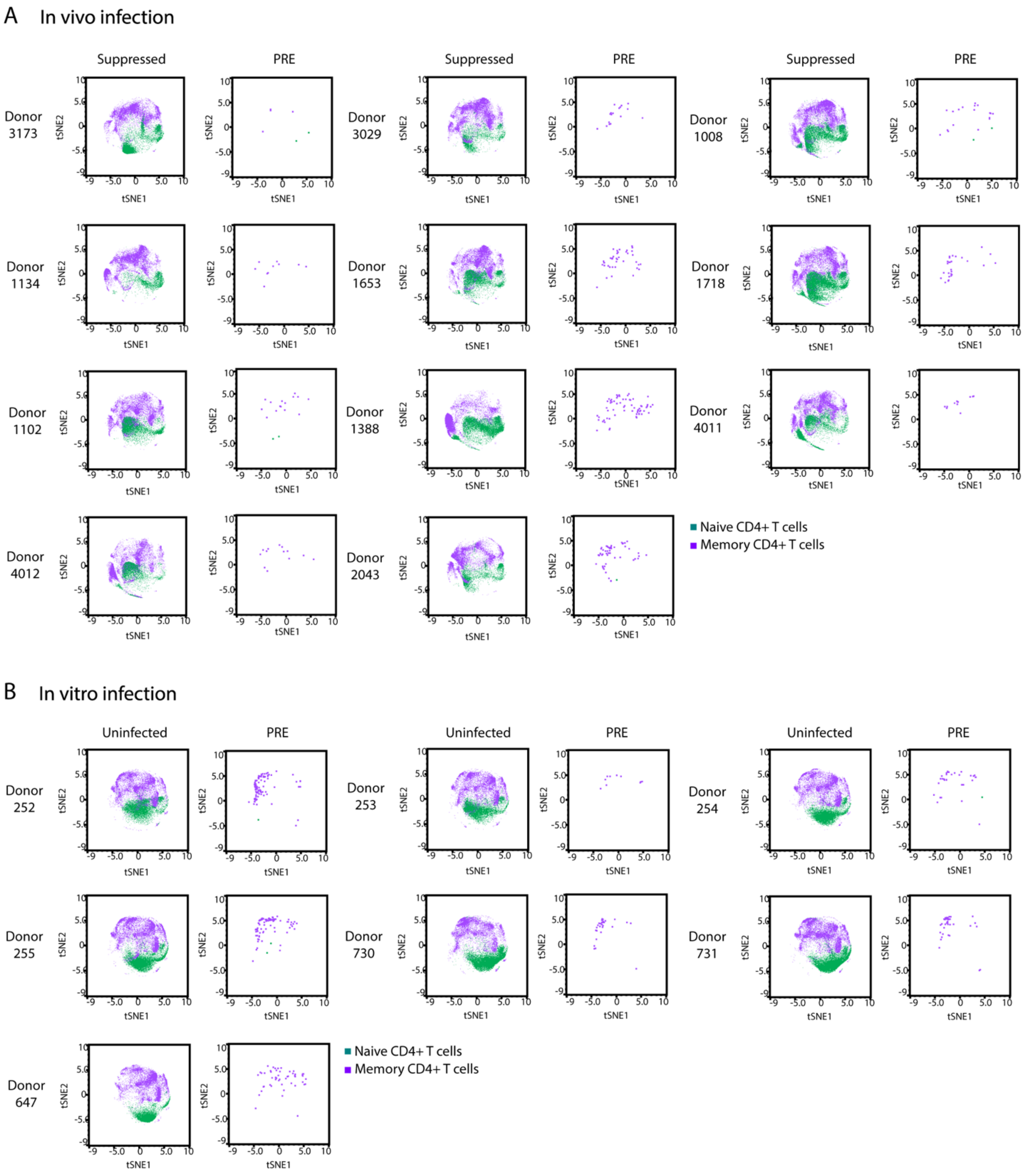

Figure S4. Memory CD4+ T cells are preferential targets of HIV both *in vivo* and *in vitro* – Related to Figure 2. tSNE plots are shown for total CD4+ T cells and PRE cells from the *in vivo* **(A)** and *in vitro* **(B)** specimens. The vast majority of PRE cells in both sets of specimens were memory (purple) and not naïve (green) cells.

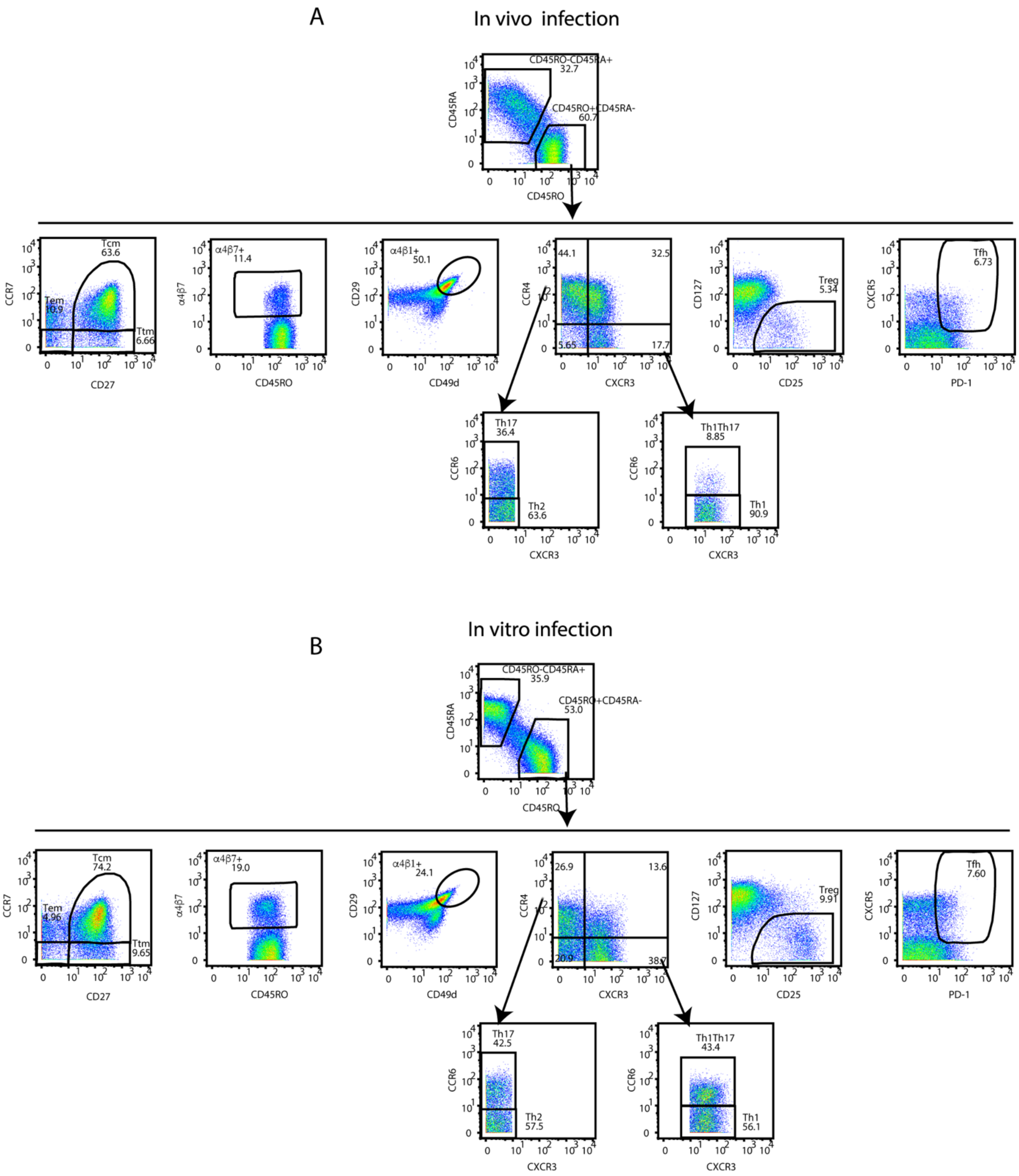

Figure S5. Gating strategies for identification of T cell subsets – Related to Figure 2.

Shown are gating strategies on representative *in vivo* **(A)** and *in vitro* **(B)** specimens. Memory CD4+ T cells were identified by gating on CD45RO+CD45RA- cells, followed by the indicated gating strategies.

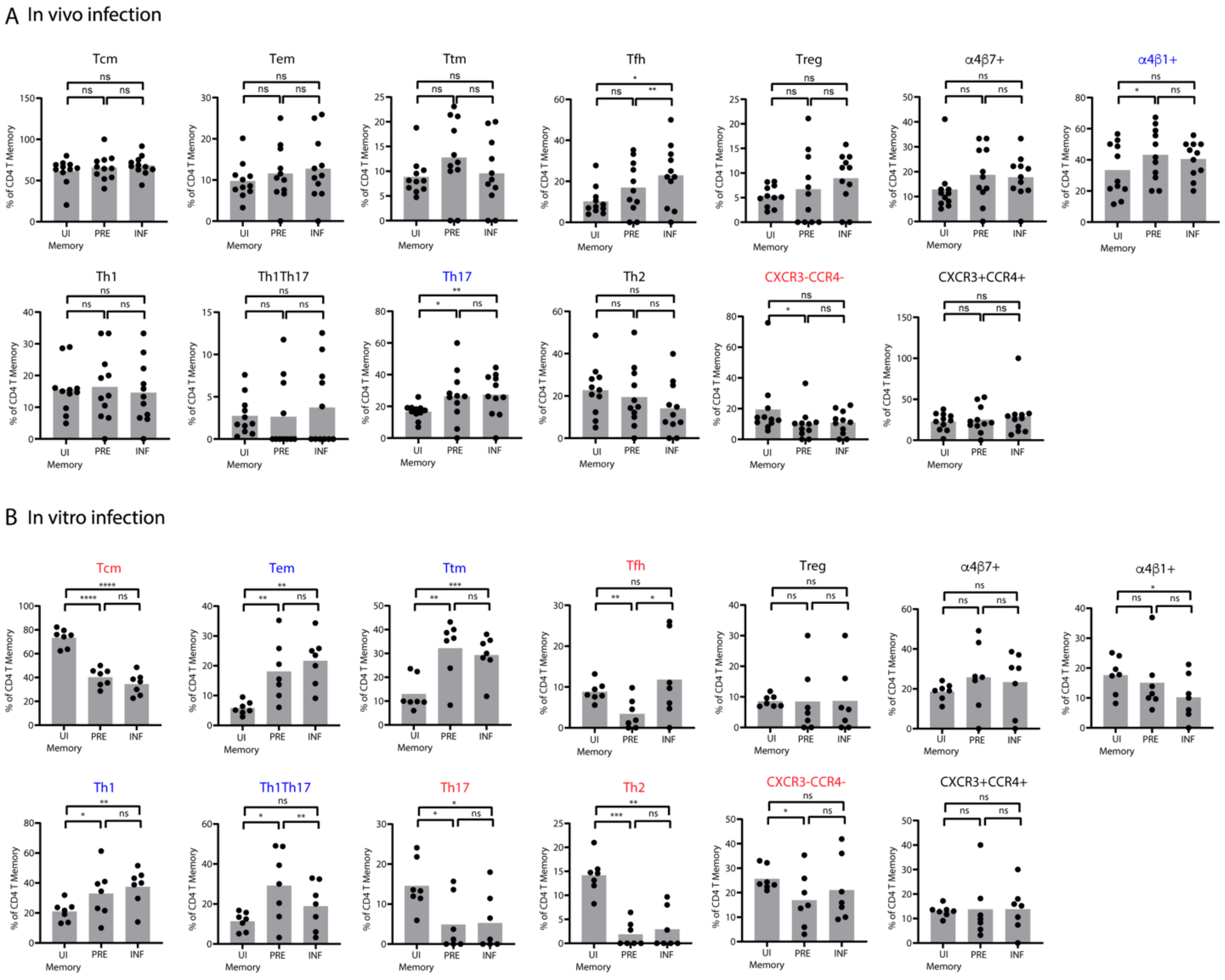

Figure S6. Frequencies of cellular subsets in UI memory, PRE, and INF specimens among memory CD4+ T cells – Related to Figure 2. All populations, including the uninfected (UI) cells, were first pre-gated on memory (CD45RO+CD45RA-) CD4+ T cells. Within this memory population, subsets were defined as follows: Tcm: CCR7+CD27+; Tem: CCR7-CD27-; Ttm: CCR7-CD27+; α4β7+ subset: Act1+; α4β1+ subset: CD29+CD49d+; Th2: CCR4+CXCR3-CCR6-; Th17: CCR4+CXCR3-CCR6+; Th1: CCR4-CXCR3+CCR6-; Th1Th17: CCR4-CXCR3+CCR6+; Treg: CD25+CD127-; Tfh: CXCR5+PD1+. The PRE and INF data are identical to those presented in Fig. 2. Datasets were from the *in vivo* **(C)** or *in vitro* **(D)** specimens. * $p < 0.05$, ** $p < 0.01$, *** $p < 0.001$ and *** $p < 0.001$ as determined by a Student's paired t-test. ns: not significant. Subsets whose frequencies are significantly higher in PRE as compared to UI

memory cells (i.e. those that are preferentially targeted for infection) are highlighted in blue, while those whose frequencies are significantly lower in PRE as compared to UI memory cells (i.e. those that are preferentially spared from infection) are highlighted in red.

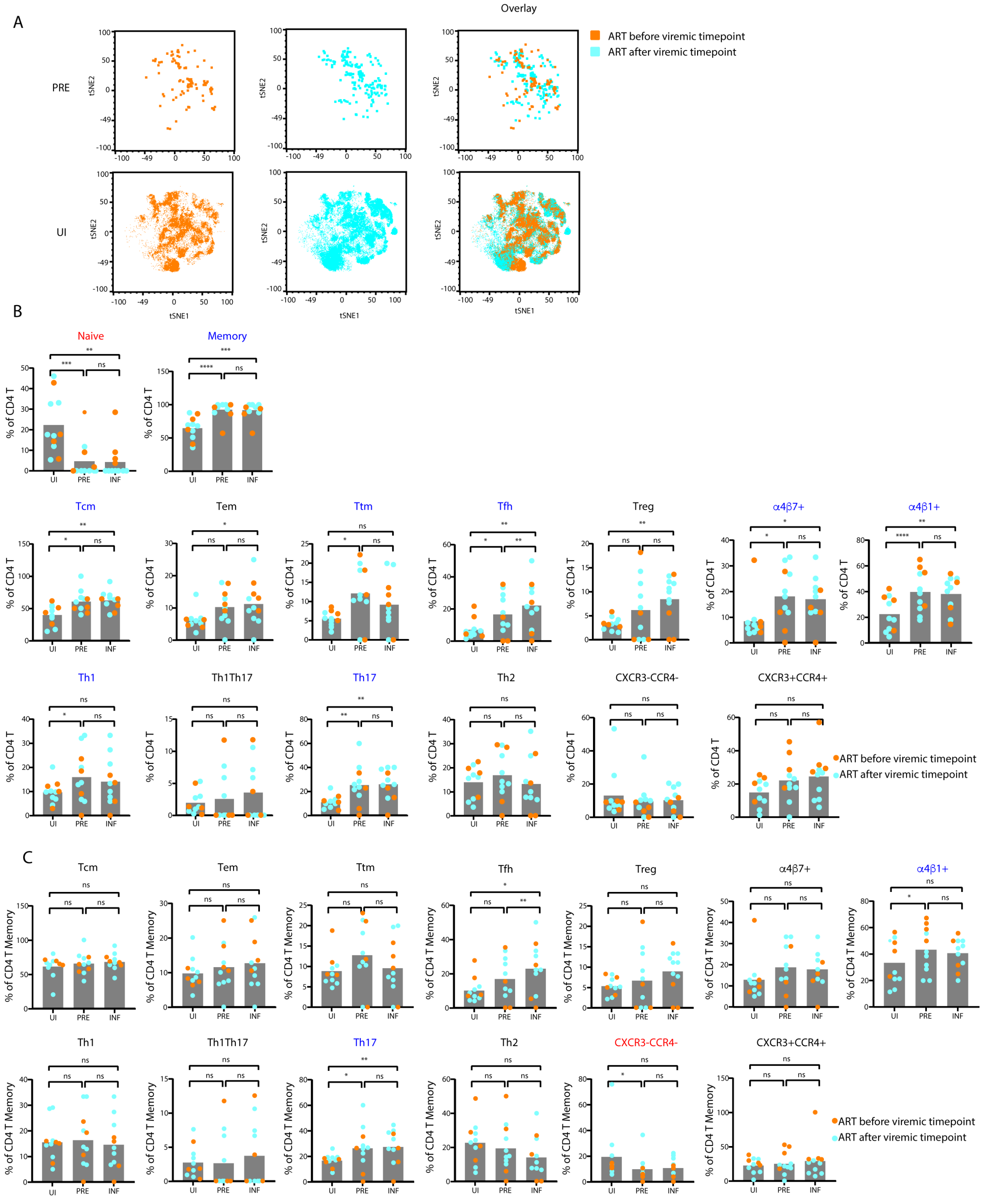

Figure S7. Uninfected, PRE, and HIV-infected CD4+ T cells are similar between the *in vivo* specimens whose ART suppression timepoint was obtained before vs. after the viremic timepoint – Related to Figure 2. A) tSNE depiction of the uninfected (UI) CD4+ T cells and

PRE cells from the *in vivo* specimens, demonstrating large overlaps between the pairs of specimens where the ART-suppressed timepoints were obtained before (*orange*) vs. after (*aqua*) viremia. **B)** Frequencies of cellular subsets in UI, PRE, and INF specimens among total CD4+ T cells. Subsets were defined as described in Fig. 2. **C)** Frequencies of cellular subsets in UI, PRE, and INF specimens among memory CD4+ T cells. Subsets were defined as described in Fig. S5. * $p < 0.05$, ** $p < 0.01$, and *** $p < 0.001$ as determined by a Student's paired t-test. ns: not significant.

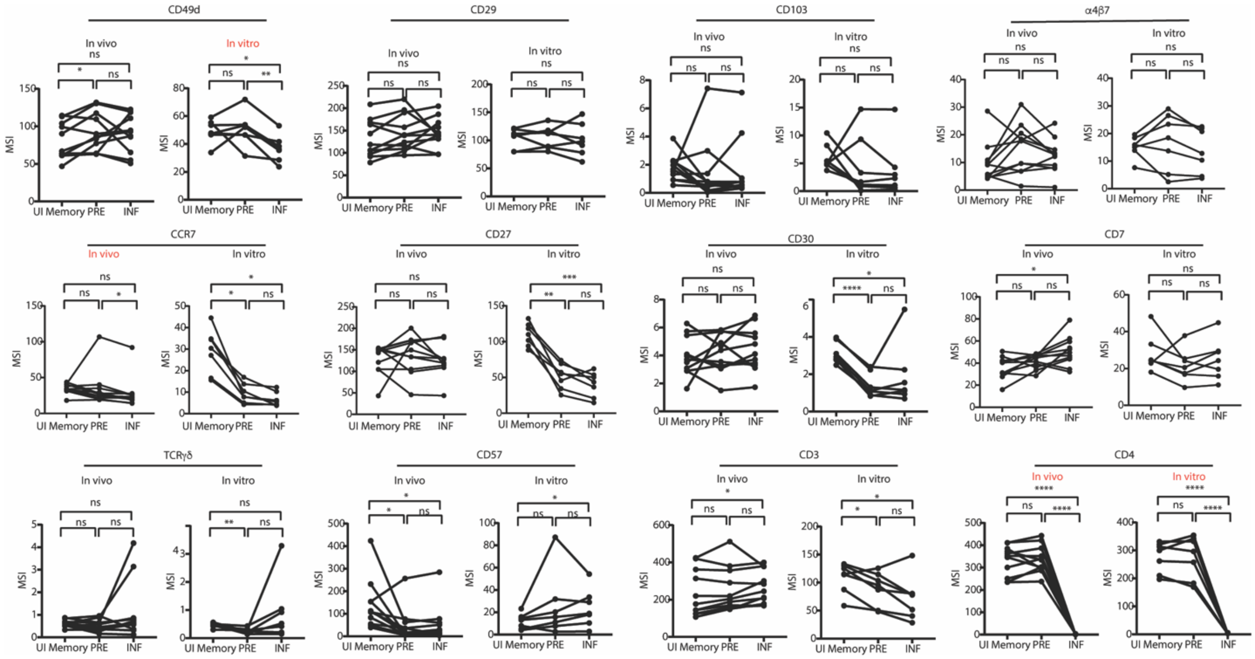

Figure S8. Surface antigens remodeled by HIV *in vivo* and *in vitro* – Related to Figure 3.

The MSIs of the phenotyping parameters not presented in Fig. 3 are presented here. Shown are MSIs of the indicated antigens on uninfected memory CD4+ T cells (UI Memory), PRE cells, and INF cells from the *in vivo* and *in vitro* specimens. * $p < 0.05$ and **** $p < 0.0001$ as determined by a Student's paired t-test and adjusted for multiple testing using the Benjamini-Hochberg for FDR. ns: not significant. Remodeled receptors whose MSIs are significantly lower in INF as compared to PRE cells are highlighted in red.

A In vivo infection

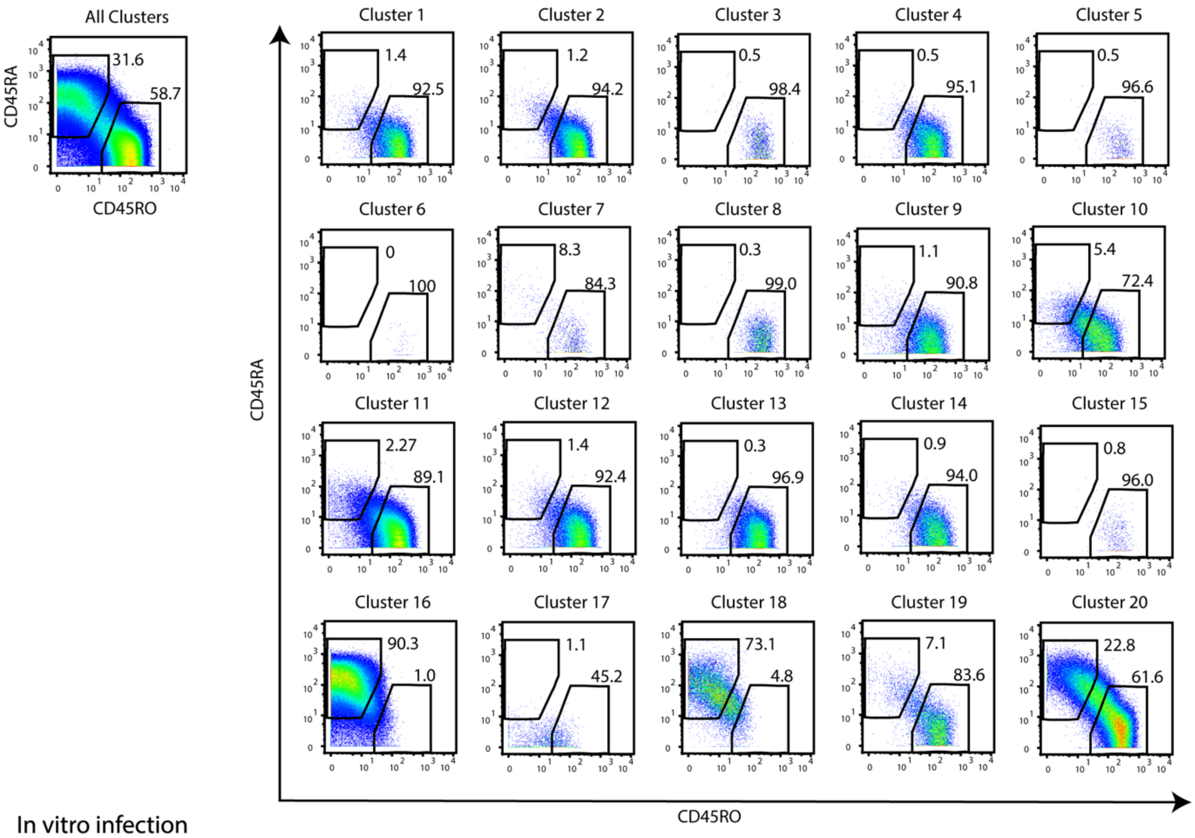

B In vitro infection

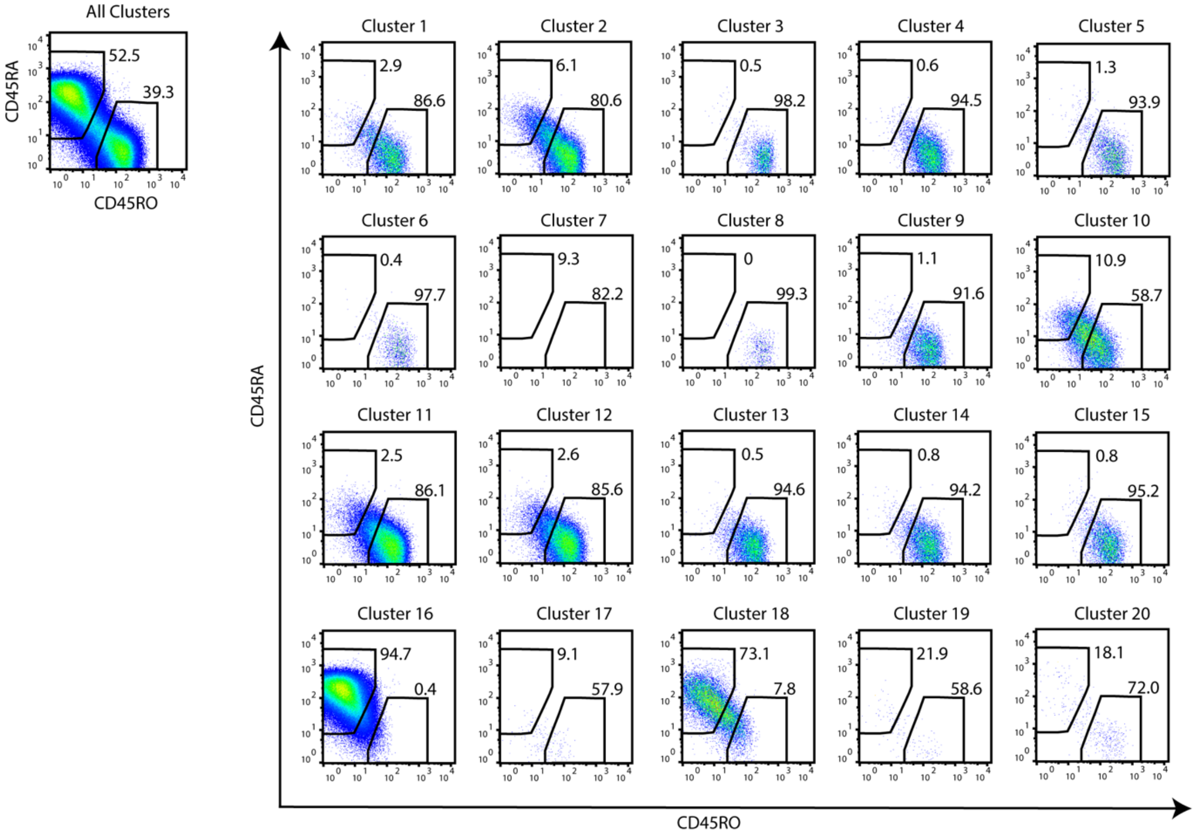

Figure S9. CD45RA and CD45RO expression on clusters of CD4+ T cells – Related to Figure 4. The proportions of CD45RA+CD45RO- and CD45RA-CD45RO+ CD4+ T cells are shown for total T cells, or for each FlowSOM-generated cluster. Naïve cells reside within the CD45RA+CD45RO-, while memory cells reside within the CD45RA-CD45RO+ population. Concatenated events corresponding to all CD4+ T cells from the *in vivo* **(A)** or *in vitro* **(B)** specimens are shown.

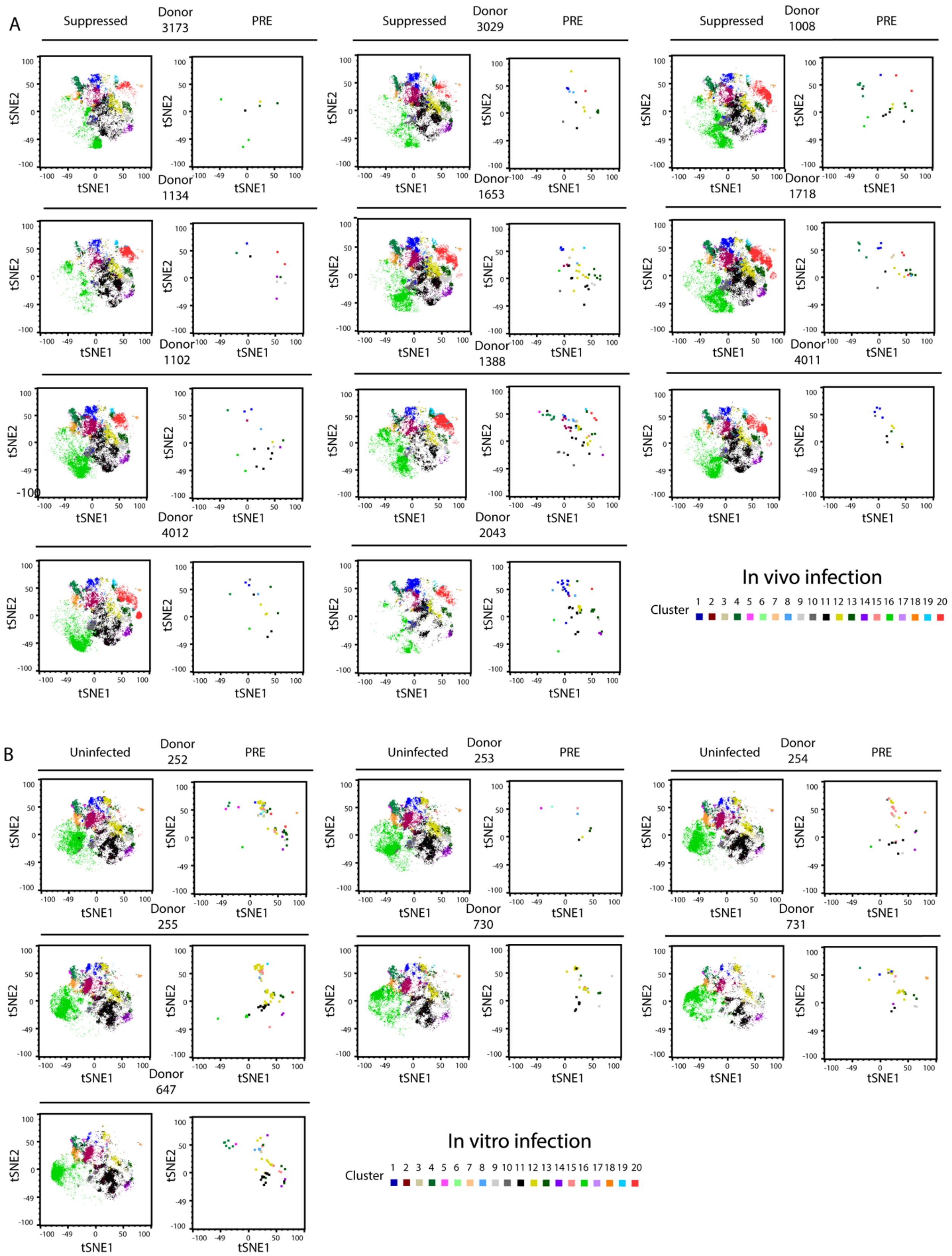
A
Suppressed
PRE
Donor 3173
Donor 3029
Donor 1008
Donor 1134
Donor 1653
Donor 1718
Donor 1102
Donor 1388
Donor 4011
Donor 4012
Donor 2043
In vivo infection
Cluster
1 2 3 4 5 6 7 8 9 10 11 12 13 14 15 16 17 18 19 20
B
Uninfected
PRE
Donor 252
Donor 253
Donor 254
Donor 255
Donor 730
Donor 731
Donor 647
In vitro infection
Cluster
1 2 3 4 5 6 7 8 9 10 11 12 13 14 15 16 17 18 19 20
tSNE1
tSNE2

Figure S10. PRE cells distribute differently from UI cells *in vivo* and *in vitro* – Related to Figure 4. tSNE depiction of all the uninfected CD4+ T cells (UI) and PRE cells from the *in vivo* (n=11) and *in vitro* (n=7) specimens, separated out by donor. Cells were clustered by FlowSOM, and colored according to the cluster to which they belonged. The difference in cluster distribution of the PRE relative to that of the UI cells suggest a non-random selection of CD4+ T cells for infection by HIV. Donors 3173, 3029, 1008, and 2043 were individuals whose viremic timepoint was subsequent to ART suppression, while donors 1134, 1653, 1178, 1102, 1388, 4011, and 4012 were individuals whose viremic timepoint was prior to ART initiation.

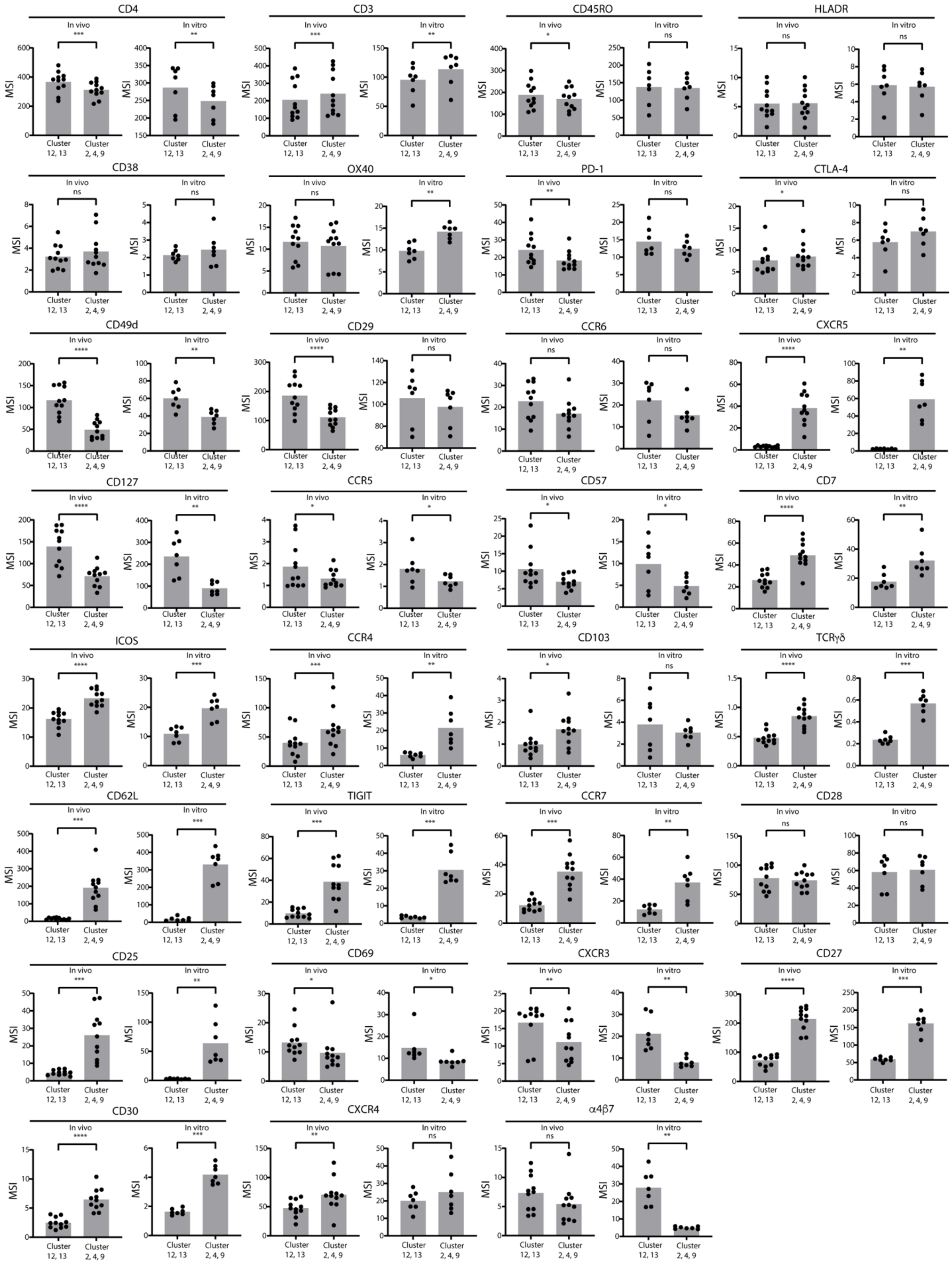
CD4
In vivo ***
In vitro **
CD3
In vivo ***
In vitro **
CD45RO
In vivo *
In vitro ns
HLADR
In vivo ns
In vitro ns
CD38
In vivo ns
In vitro ns
OX40
In vivo ns
In vitro **
PD-1
In vivo **
In vitro ns
CTLA-4
In vivo *
In vitro ns
CD49d
In vivo ****
In vitro **
CD29
In vivo ****
In vitro ns
CCR6
In vivo ns
In vitro ns
CXCR5
In vivo ****
In vitro **
CD127
In vivo ****
In vitro **
CCR5
In vivo *
In vitro *
CD57
In vivo *
In vitro *
CD7
In vivo ****
In vitro **
ICOS
In vivo ****
In vitro ***
CCR4
In vivo ***
In vitro **
CD103
In vivo *
In vitro ns
TCRγδ
In vivo ****
In vitro ***
CD62L
In vivo ***
In vitro ***
TIGIT
In vivo ***
In vitro ***
CCR7
In vivo ***
In vitro **
CD28
In vivo ns
In vitro ns
CD25
In vivo ***
In vitro **
CD69
In vivo *
In vitro *
CXCR3
In vivo **
In vitro **
CD27
In vivo ****
In vitro ***
CD30
In vivo ****
In vitro ***
CXCR4
In vivo **
In vitro ns
α4β7
In vivo ns
In vitro **
MSI
Cluster 12, 13
Cluster 2, 4, 9

Figures S11. Comparison of antigen expression in preferentially susceptible vs. preferentially resistant clusters of memory T cells – Related to Figure 4. The cell population consisting of HIV-susceptible clusters 12 and 13 was compared to the cell population consisting of clusters 2, 4, and 9 which were preferentially spared from infection. Each datapoint corresponds to a different donor. * $p < 0.05$, ** $p < 0.01$, *** $p < 0.001$, and **** $p < 0.0001$ as determined by a Student's paired t-test and adjusted for multiple testing using the Benjamini-Hochberg for FDR. ns: not significant.

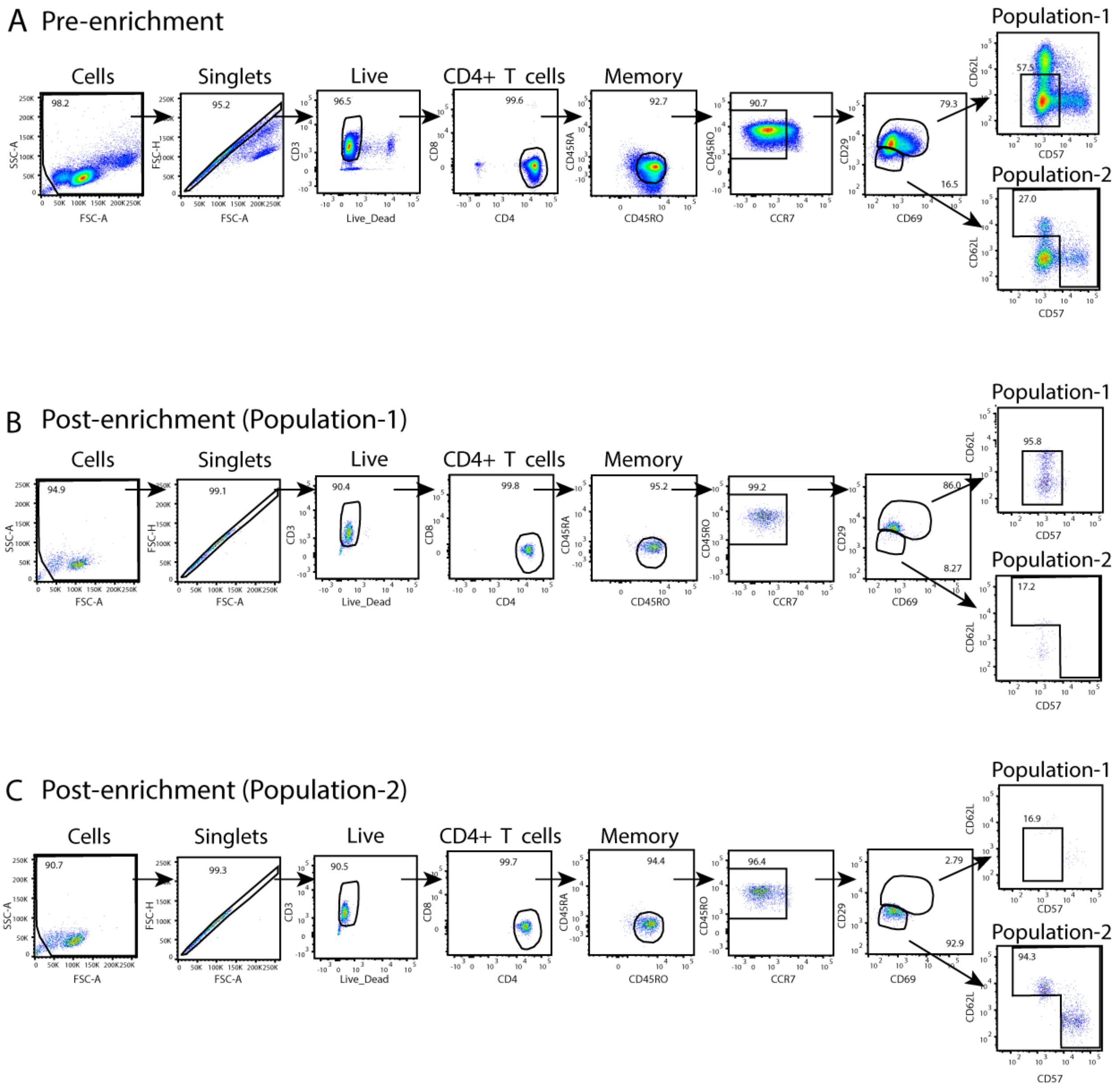

Figure S12. Gating strategy for sorting of Population-1 and Population-2 cell populations – Related to Figure 7. Memory CD4+ T cells were purified by bead-based negative selection prior to sorting to minimize sorting time, and then sorted on an Aria II instrument. **(A)** Sorting strategy to isolate Population-1 (CD3+CD4+CD45RO+CD45RA-CCR7$^{low/med}$CD29$^{med/high}$CD69$^{med/high}$CD62L^{low}CD57$^{low/med}$) and Population-2

(CD3+CD4+CD45RO+CD45RA-CCR7$^{low/med}$CD29^{low}CD69^{low} and not CD62L^{low}CD57$^{low/med}$) cells.

Post-sort purities of Population-1 **(B)** and Population-2 **(C)** are also shown.
